# Construction of Bio‐Piezoelectric Platforms: From Structures and Synthesis to Applications

**DOI:** 10.1002/adma.202008452

**Published:** 2021-05-25

**Authors:** Qianqian Xu, Xinyu Gao, Senfeng Zhao, You‐Nian Liu, Dou Zhang, Kechao Zhou, Hamideh Khanbareh, Wansong Chen, Yan Zhang, Chris Bowen

**Affiliations:** ^1^ Hunan Provincial Key Laboratory of Micro & Nano Materials Interface Science College of Chemistry and Chemical Engineering Central South University Hunan 410083 China; ^2^ State Key Laboratory of Powder Metallurgy Central South University Hunan 410083 China; ^3^ Department of Mechanical Engineering University of Bath Bath BA27AY UK

**Keywords:** biomedicine, biosensing, disease treatment, piezoelectric materials

## Abstract

Piezoelectric materials, with their unique ability for mechanical‐electrical energy conversion, have been widely applied in important fields such as sensing, energy harvesting, wastewater treatment, and catalysis. In recent years, advances in material synthesis and engineering have provided new opportunities for the development of bio‐piezoelectric materials with excellent biocompatibility and piezoelectric performance. Bio‐piezoelectric materials have attracted interdisciplinary research interest due to recent insights on the impact of piezoelectricity on biological systems and their versatile biomedical applications. This review therefore introduces the development of bio‐piezoelectric platforms from a broad perspective and highlights their design and engineering strategies. State‐of‐the‐art biomedical applications in both biosensing and disease treatment will be systematically outlined. The relationships between the properties, structure, and biomedical performance of the bio‐piezoelectric materials are examined to provide a deep understanding of the working mechanisms in a physiological environment. Finally, the development trends and challenges are discussed, with the aim to provide new insights for the design and construction of future bio‐piezoelectric materials.

## Graphical abstract

Bio‐piezoelectric platforms with the ability for mechanical–electrical energy conversion exhibit great potential in the biomedical field. The latest developments in bio‐piezoelectric platforms are introduced, their design and engineering strategies are highlighted, and the current state‐of‐the‐art in biomedical applications is systematically outlined. One closely combines the range of materials structures with their biomedical performance, aiming to provide insights for their design and construction in the future.

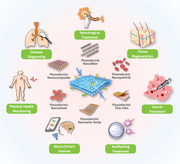

## 1 Introduction

The piezoelectric effect was first discovered and demonstrated in 1880 by Curie brothers, citing the Greek word for “pressure electricity.”^[^ ^1^ ^]^ As the name suggests, the piezoelectric effect is characterized by a linear transformation between mechanical and electrical variables.^[^ ^2^ ^]^ In general, piezoelectric materials can generate charges on two opposing surfaces in response to a mechanical strain, such as stretching, compression, or bending. From the perspective of crystallography, the piezoelectric effect is ascribed to the asymmetry of crystal structures or molecular chains. When ions with different charges are asymmetrically arranged in piezoelectric crystals, electric dipole moments are formed. Ferroelectric materials represent a sub‐class of piezoelectric materials piezoelectric materials where their electric dipole moments are uniformly oriented in specific regions, leading to the formation of domain structures. Since the electric domains are randomly distributed in crystals, the polarization would be mutually counter‐balanced and the overall polarization intensity of the material is zero. A unique aspect of ferroelectric materials is that upon exposure to a large external electric field, the domain orientation can be aligned in a single direction to achieve an overall material polarization to enhance the piezoelectric response.^[^ ^3^ ^]^

When piezoelectric materials are subjected to an external stress, the distance between positive and negative charge centers is changed, resulting in material polarization along the stress direction.^[^ ^3^, ^4^ ^]^ In this case, the surface free charges are partially released to generate piezoelectricity, which is termed the direct piezoelectric effect. Likewise, under the action of an external electric field, piezoelectric materials can produce a geometric deformation proportional to the external electric field, which is termed the converse piezoelectric effect. Representative piezoelectric materials include nonferroelectric materials such as quartz and zinc oxide (ZnO),^[^ ^5^ ^]^ and ferroelectric materials such as barium titanate (BaTiO_3_),^[^ ^6^ ^]^ lead zirconate titanate (Pb[Zr*_x_* Ti_1−_ *_x_*]O_3_, PZT),^[^ ^7^ ^]^ lithium niobate (LiNbO_3_),^[^ ^8^ ^]^ potassium sodium niobate (K_0.5_Na_0.5_NbO_3_, KNN),^[^ ^9^ ^]^ polyvinylidene fluoride (PVDF) and its copolymers.^[^ ^10^ ^]^ Interestingly, some animal tissues, such as collagen^[^ ^11^ ^]^ and hydroxyapatite,^[^ ^12^ ^]^ have also been found to be piezoelectric, which is a result of the asymmetrical structure of biological molecules, such as amino acids, that leads to the formation of a dipole.

**Figure 1:**
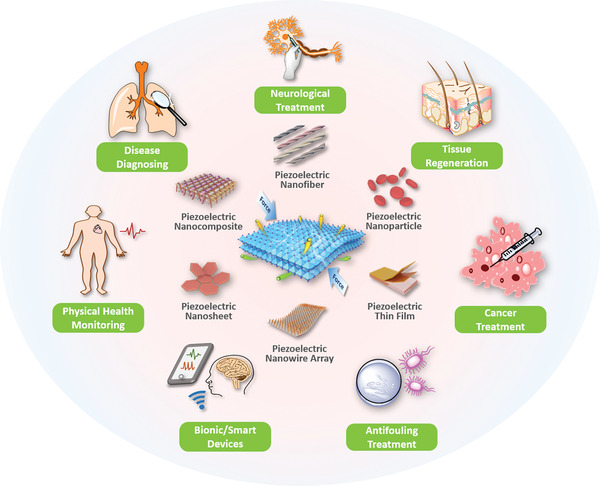
Schematic of the diverse range of bio‐piezoelectric platforms, and their biomedical applications.

Since their discovery, piezoelectric materials have been widely applied in sensing, actuation, energy conversion,^[^ ^4^, ^13^ ^]^ and more recently catalysis.^[^ ^14^ ^]^ In recent years, with the identification of biocompatible piezoelectric and ferroelectric materials, such as BaTiO_3_ and KNN,^[^ ^15^ ^]^ the transformation between mechanical energy and electrical energy has been successfully achieved in biological systems,^[^ ^16^ ^]^ which opens the door to the development of new bio‐piezoelectric materials for a range of biomedical applications. For example, by virtue of surface modification and material engineering strategies, piezoelectric materials can become functionalized bio‐piezoelectric materials^[^ ^3^, ^12^ ^]^ that are able to connect and interact with human tissues (such as skin, muscles, bones, organs, cells, and nerves) for a range of biomedical applications. To date, there have been examples of excellent reviews regarding the progress of piezoelectric materials as sensors,^[^ ^3^, ^17^ ^]^ energy harvesters,^[^ ^2^, ^18^ ^]^ biocompatible devices,^[^ ^1^, ^19^ ^]^ smart textiles,^[^ ^20^ ^]^ tissue regeneration,^[^ ^21^ ^]^ and electronic skins.^[^ ^22^ ^]^ In this review, we present bio‐piezoelectric platforms from a broad perspective, with a focus on their manufacture and design strategies. We systematically outline the representative structures of bio‐piezoelectric materials, with an emphasis on their synthesis and engineering methods. The latest biomedical applications of bio‐piezoelectric platforms in terms of health monitoring, disease diagnosis, bionic/smart devices, cancer treatment, tissue regeneration, neurotrauma treatment, and antifouling treatment are summarized. These topics are outlined in **Figure** **1**, where the review aims to provide a deep understanding of the working mechanisms of state‐of‐the‐art piezoelectric platforms and inspire new insights for future design strategies. Finally, the future challenges and opportunities related to bio‐piezoelectric systems in biomedical applications are discussed.

## 2 Structures and Materials

To meet the critical requirements of biomedical applications, piezoelectric materials should possess high electromechanical coupling and good biocompatibility. There may also be a need to be sensitive to stimuli, be able respond to physiological behavior and offer high mechanical flexibility and strength.^[^ ^23^ ^]^ From the perspective of their chemical structure, current piezoelectric materials for bio‐piezoelectric platforms can be broadly classified into four categories: i) bio‐piezoelectric ceramics, ii) bio‐piezoelectric polymers, iii) biomolecular piezoelectric materials, and iv) bio‐piezoelectric nanomaterials.

### 2.1 Bio‐Piezoelectric Ceramics

Polycrystalline ferroelectric ceramics possess randomly oriented domains, where each domain presents a disordered polarization vector. To provide the ceramics with a macroscopic piezoelectric effect, it is necessary to polarize them by applying a strong electric field above its coercive field, thereby allowing the polarization vectors to rearrange along the direction of electric field. In general, piezoelectric properties of ceramics are closely associated with their crystal structures, as discussed below.

**Figure 2:**
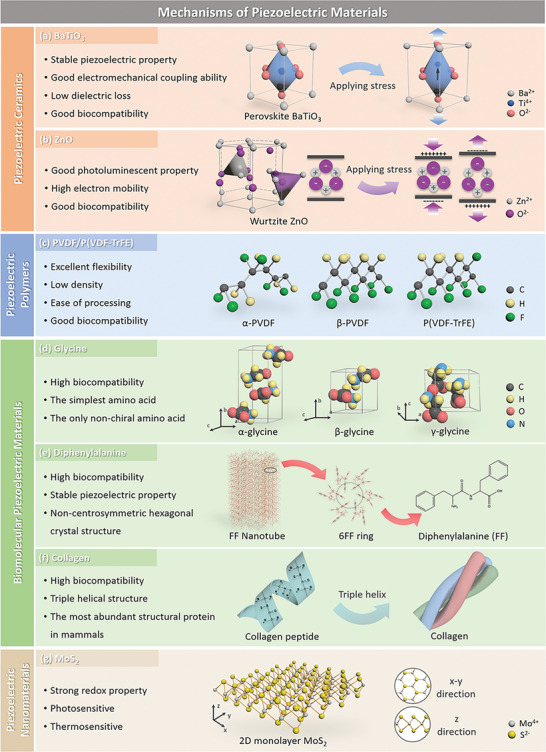
Characteristics of the bio‐piezoelectric materials and their atomic structures: a) perovskite BaTiO_3_, b) wurtzite ZnO, c) PVDF/P(VDF‐TrFE), d) glycine, e) diphenylalanine (FF) peptide nanotube, f) collagen, and g) 2D monolayer MoS_2_.

A number of ferroelectric materials exhibit the perovskite crystal structure, which belongs to a cubic crystal system. The general chemical formula of a perovskite is ABO_3_,^[^ ^24^ ^]^ where A represents a lanthanide or alkali earth metal, and B refers to a transition metal. Both O and A, with their large ionic radii, are in a cubic close packed configuration, while B has a smaller ionic radius to fill the central void of the octahedron. As a typical perovskite type bio‐piezoelectric material, PZT has been commonly used in sensing, catalysis, and energy harvesting due to their high piezoelectric activity, high electromechanical coupling and low manufacturing cost. Since PZT contains lead (Pb), it is potentially toxic to living organisms^[^ ^3^ ^]^ and its application in biological systems is seriously restricted. To overcome this limitation, a number of lead‐free ferroelectric materials with a perovskite structure have been developed.^[^ ^25^ ^]^ In particular, BaTiO_3_ features an improved biocompatibility, good electromechanical coupling,^[^ ^26^ ^]^ and has been proven to be a promising candidate for a variety of biomedical applications.^[^ ^27^ ^]^ Structural analysis reveals that BaTiO_3_ is a non‐centrosymmetric tetragonal crystal and when subjected to an external mechanical stress, the BaTiO_3_ crystal exhibits a strong electrical polarity due to the shift of Ti^4+^ within the tetragonal unit cell; as seen in **Figure** **2a**. In addition to BaTiO_3_, other lead‐free ferroelectric ceramics with the perovskite structure have been developed, including BiFeO_3_, LiNbO_3_, KNN, alkali niobate (K, Na, Li)NbO_3,_ and alkaline bismuth titanate (K, Na)_0.5_Bi_0.5_TiO_3_.^[^ ^25^, ^28^ ^]^

A second common piezoelectric crystal structure is wurtzite that belongs to a hexagonal crystal system with a tetrahedral coordination AB‐type composition. In this type of crystal structure, the A atoms are in hexagonal close‐packed arrangement with B atoms occupying the tetrahedral void. Many nonferroelectric bio‐piezoelectric materials possess the wurtzite crystal structure, such as ZnO, AlN, GaN, InN, CdS, and CdSe.^[^ ^29^ ^]^ Since their polarization direction cannot be changed by a poling field, these materials are used in single crystal or highly textured form along specific crystallographic directions. In the ZnO crystal structure, the Zn^2+^ and O^2−^ are stacked layer by layer along the c‐axis with their anion and cationic charge centers coinciding with each other.^[^ ^2^ ^]^ When subjected to an external force, the positive and negative charge centers separate from each other to generate a piezoelectric potential, as shown in Figure 2b. In addition, ZnO also exhibits a high electron mobility, good transparency, and strong photoelectric properties. When combined with its piezoelectric properties, ZnO can be used in a variety of optical imaging systems,^[^ ^30^ ^]^ energy collection devices,^[^ ^31^ ^]^ or piezoelectric sensors.^[^ ^32^ ^]^ Moreover, the excellent biocompatibility of ZnO makes it attractive candidate in biological imaging probes and tissue engineering scaffolds.^[^ ^30^, ^33^ ^]^ Similarly, wurtzite‐based AlN is another popular bio‐piezoelectric material for biomedical applications due to its low toxicity. The wide bandgap and strong electromechanical coupling capability of AlN also allow it to be widely applied in sensors and resonator devices.^[^ ^3^, ^34^ ^]^

Despite their outstanding piezoelectric properties, most bio‐piezoelectric ceramics suffer from some inherent shortcomings, such as the need for high temperature processing, high rigidity, and brittleness. As a result, bio‐piezoelectric ceramics exhibit a poor mechanical tolerance to defects and externally applied strains. Most bio‐piezoelectric sensors and transducers, such as wearable biosensors or implantable devices, often place high demands on the flexibility of the materials. As a result, the low fracture toughness of bio‐piezoelectric ceramic materials makes them susceptible to fracture when subject to mechanical loads or flexure, which restricts their biomedical applications. In order to counteract this deficiency, bio‐piezoelectric ceramics have been assembled into ultra‐thin films to reduce the defect size and improve mechanical flexibility.^[^ ^35^ ^]^

### 2.2 Bio‐Piezoelectric Polymers

Due to their asymmetrical molecular structure and orientation, some flexible polymers exhibit ferroelectricity and piezoelectricity by stretching, which results in an electrical polarization via molecular dipole reorientation in the bulk polymer.^[^ ^3^, ^36^ ^]^ Bio‐piezoelectric polymers are attractive bio‐piezoelectric materials due to their excellent mechanical flexibility, light weight nature, low dielectric constant, and ease of processing at low temperatures.^[^ ^37^ ^]^ The electromechanical coupling efficiency and optical transparency of the bio‐piezoelectric polymers can be finely tailored through the processing parameters.^[^ ^18a^ ^]^ More importantly, most bio‐piezoelectric polymers are biocompatible with minimal toxicity, ^[^ ^38^ ^]^ and bio‐piezoelectric polymers such as poly‐β‐hydroxybutyrate and poly‐3‐hydroxybutyrate‐3‐hydroxy valerate are even biodegradable in the human body. These advantages make bio‐piezoelectric polymers promising candidates for the creation of miniature, implantable and flexible electronic devices. In recent years, the development of functional bioimplants in sensing and actuation has stimulated a demand for bio‐piezoelectric polymer materials.^[^ ^1^ ^]^

PVDF is one of the most common bio‐piezoelectric polymers composed of —CH_2_CF_2_— monomers. Its molecular dipoles are generated from the difference in electronegativity between hydrogen and fluorine atoms.^[^ ^37^ ^]^ In general, PVDF exhibits five forms of crystalline phase (α, β, γ, δ, ε), among which the α‐ and β‐ phases are the most common, as shown in Figure 2c.^[^ ^3^, ^39^ ^]^ The α‐phase of PVDF consists of dipoles in a reverse parallel order, thereby showing little piezoelectricity. In contrast, the dipoles in the β‐phase are arranged in parallel, providing a high dipole moment per unit cell and superior piezoelectric properties.^[^ ^40^ ^]^ To further improve the performance of PVDF material, a variety of PVDF copolymers have been developed, such as polyvinylidene fluoride trifluoro ethylene [P(VDF‐TrFE)], see Figure 2c.^[^ ^41^ ^]^ Compared with PVDF, the P(VDF‐TrFE) copolymer exhibits superior crystallinity, higher flexibility, higher residual polarization and electromechanical coupling factor, making it more applicable for flexible biosensors or tissue engineering scaffolds.^[^ ^10^, ^42^ ^]^ Moreover, the high strength of the C—F bonds allows the P(VDF‐TrFE) copolymer to stabilize and preserve its piezoelectricity when exposed to biological environments, or electromagnetic radiation in the form of ultraviolet light and gamma rays, that is often used for sterilization.^[^ ^10^ ^]^

In addition to PVDF and its copolymers, the piezoelectric effect has been found in other polymers, such as poly‐l‐lactic acid,^[^ ^43^ ^]^ polyacrylonitrile,^[^ ^44^ ^]^ poly‐β‐hydroxybutyrate,^[^ ^45^ ^]^ polyvinyl chloride,^[^ ^46^ ^]^ and odd‐numbered nylon (e.g., nylon‐11).^[^ ^47^ ^]^ If we consider nylon‐11 as an example, the arrangement of polyamide molecules in adjacent nylon‐11 chains during crystallization results in multiple‐hydrogen bonding and dipole orientation. In this regard, nylon‐11 exhibits a high melting point and comparable piezoelectricity to PVDF.^[^ ^48^ ^]^ Furthermore, as a typical bio‐piezoelectric polymer, nylon‐11 exhibits excellent mechanical flexibility, good fatigue resistance, and piezoelectric performance without the need for polarization using high electric fields.^[^ ^49^ ^]^ These advantages make nylon‐11 based bio‐piezoelectric polymers attractive candidates as piezoelectric fibers for electronic textiles and bio‐piezoelectric platforms.

It is worth noting that most bio‐piezoelectric polymers usually exhibit relatively low piezoelectric charge coefficients (*d_ij_*) compared to the inorganic bio‐piezoelectric materials described above, leading to lower levels of charge generation.^[^ ^50^ ^]^ To improve piezoelectric performance, bio‐piezoelectric polymers have been integrated with inorganic bio‐piezoelectric materials to construct bio‐piezoelectric composites. Compared with individual piezoelectric components, bio‐piezoelectric composites can overcome the temperature limitation of bio‐piezoelectric polymers and the inherent brittleness of inorganic bio‐piezoelectric materials while providing ease of manufacturing for large‐area applications.^[^ ^51^ ^]^ For example, bio‐piezoelectric polymers have been successfully combined with bio‐piezoelectric ceramics, achieving synergistically enhanced piezoelectricity, biocompatibility, and mechanical flexibility.^[^ ^51^, ^52^ ^]^ Therefore, bio‐piezoelectric composites provide a compelling alternative to conventional piezoelectric materials for biomedical applications.^[^ ^53^ ^]^

### 2.3 Biomolecular Piezoelectric Materials

The piezoelectric effect is also found in many biomolecules (e.g., amino acids, peptides, and proteins) and biological tissues (bones, ligaments, tendons, skins, and hairs),^[^ ^12b^ ^]^ collectively termed biomolecular piezoelectric materials. These materials are attractive for the biomedical field as a result of their high biocompatibility, stable piezoelectric coefficients, and dielectric properties.^[^ ^12^, ^54^ ^]^ When subjected to mechanical stimuli, biomolecular piezoelectric materials generate surface charge polarization or electric field, both of which have been demonstrated with appropriate physiological functions, such as tissue growth, wound healing, and regeneration.^[^ ^55^ ^]^

As a basic unit of biomolecules, amino acids are composed of carboxyl group (—COOH), amino group (—NH_2_) and variable side chains, all of which are attached to a central carbon atom. The difference between various amino acids depends on the structure of their side chains. Taking glycine as an example, under different crystallization conditions, glycine forms three kinds of crystal structures, namely α, β, and γ (Figure 2d).^[^ ^56^ ^]^ The α‐glycine crystal exhibits crystallographic symmetry and therefore lacks piezoelectricity, while both the β‐glycine and γ‐glycine have non‐centrosymmetric crystal structures and exhibit ferroelectric properties.^[^ ^12^, ^57^ ^]^ Recent studies reveal that the piezoelectric charge coefficient of β‐glycine, a measure of the charge per unit force or strain per unit electric field, can reach approximately 10 pm V^−1^, which is comparable to traditional organic piezoelectric materials.^[^ ^12^, ^57^ ^]^

With amino acids as the basic unit, peptides and proteins are constructed with a variety of structures and functions. The amino acid sequence and spatial configuration ultimately determine the biological function of peptides and proteins, thus providing structure‐dependent piezoelectric properties.^[^ ^12b^ ^]^ For example, diphenylalanine is a dipeptide composed of two phenylalanine, which can further self‐assemble into nanostructures, such as nanotubes and hydrogels.^[^ ^58^ ^]^ The self‐assembled diphenylalanine nanotube, with its noncentrosymmetric hexagonal structure, exhibits piezoelectricity; this can be seen in Figure 2e.^[^ ^59^ ^]^ Likewise, piezoelectricity is also found in proteins, especially in collagen, due to their asymmetric spatial structures. Collagen is a triple helix structure formed by three twisted polypeptide chains, with abundant polar and charged groups in the backbone, see Figure 2f.^[^ ^60^ ^]^ When subjected to an external mechanical stress, the dipole moments of these amino acid residues in collagen reorient along the longitudinal direction, thereby leading to a change in polarization and a piezoelectric response.^[^ ^12^, ^60^, ^61^ ^]^

In addition to above‐mentioned biomolecules, specific biological tissues, such as ligaments and tendons, also exhibit the piezoelectric effect due to the presence of piezoelectric protein molecules. Likewise, the piezoelectric effect has been found in some plant tissues.^[^ ^62^ ^]^ For example, lignocellulosic molecules, which are present in many plants also exhibit a piezoelectric response, which is the origin of the piezoelectric effect in wood.^[^ ^63^ ^]^ In this regard, nanoscale cellulose molecules can be used to manufacture lightweight films and nanoscale paper, which have potential to be a future bio‐piezoelectric material for biosensors, actuators, and other biocompatible devices.^[^ ^64^ ^]^

### 2.4 Bio‐Piezoelectric Nanomaterials

Advances in nanotechnology have opened new paradigms for the development of bio‐piezoelectric nanomaterials. In general, bio‐piezoelectric nanomaterials provide merits for biomedical applications in comparison to their bulk counterparts. The ultra‐small size of bio‐piezoelectric nanomaterials allows them to efficiently traverse a variety of physiological barriers, such as blood vessels or cell membranes. Moreover, for piezo‐catalysis based biomedical applications, piezoelectric nanocatalysts often exhibit superior catalytic efficiency over bulk catalysts, since their smaller dimensions provides them with enhanced electron transfer rate and stronger interactions with any substrate. In addition, the high surface area of bio‐piezoelectric nanomaterials enables them to serve as multi‐functional drug nanocarriers for drug delivery and disease therapy. Therefore, bio‐piezoelectric nanomaterials with both piezoelectric properties and nanosize effects exhibit significant potential for a wide range of biomedical applications. In general, according to their dimension (D), bio‐piezoelectric nanomaterials can be divided into three groups: 0D, 1D and 2D.

0D bio‐piezoelectric nanomaterials generally refer to nanoparticles, nanoclusters, and quantum dots.^[^ ^21^, ^65^ ^]^ The characteristics of 0D bio‐piezoelectric nanomaterials include large surface area, excellent piezoelectric properties and being intrinsically single domain.^[^ ^66^ ^]^ Moreover, some 0D bio‐piezoelectric nanomaterials, such as BaTiO_3_ nanoparticles,^[^ ^27c^ ^]^ exhibit a high biocompatibility and fast metabolic rates to meet the critical requirements of the biomedical field.^[^ ^26^ ^]^ In addition, 0D bio‐piezoelectric nanomaterials can integrate with other bio‐piezoelectric materials, such as polymer films, to achieve improved piezoelectric properties. The advantages of 0D bio‐piezoelectric nanomaterials allow them to be employed in a number of versatile biomedical applications, such as biocatalysis^[^ ^67^ ^]^ and disease treatment.^[^ ^27a,c^ ^]^

1D bio‐piezoelectric nanomaterials are often used in the form of nanowires, nanobelts, nanotubes, nanorods, and nanofibers. Compared with 0D bio‐piezoelectric nanomaterials, 1D bio‐piezoelectric nanomaterials exhibit a higher charge transfer efficiency due to their wire‐like morphology.^[^ ^68^ ^]^ In addition, they can overcome the shortcomings of agglomeration that exists for many 0D bio‐piezoelectric nanomaterials.^[^ ^32^, ^69^ ^]^ As a result, 1D bio‐piezoelectric nanomaterials exhibit good processability, exceptional piezoelectric effects, high sensitivity, and good flexibility. Many 1D bio‐piezoelectric nanomaterials, such as PVDF ^[^ ^32^ ^]^ and ZnO nanowires, have been successfully used as electromechanical conversion components in piezoelectric biosensing or energy harvesting devices.^[^ ^70^ ^]^ If we consider PVDF nanowires as an example, their excellent mechanical flexibility enables them to withstand a high degree of strain and achieve a long operational lifetime.^[^ ^32^ ^]^ In particular, 1D bio‐piezoelectric nanomaterials can be easily integrated into piezoelectric nanowire arrays to achieve enhanced piezoelectric performance. It is worth highlighting that most 1D bio‐piezoelectric nanomaterials possess good biocompatibility, allowing them to be widely used in biosensors,^[^ ^3^ ^]^ smart textiles,^[^ ^20^ ^]^ and electronic skins.^[^ ^22^ ^]^

When thinned down to a nanometer thickness to create a 2D geometry, piezoelectric materials and even conventional non‐piezoelectric materials, can lose their centrosymmetry in one direction and exhibit an enhanced level of piezoelectricity.^[^ ^66^, ^71^ ^]^ The obtained 2D bio‐piezoelectric nanomaterials are planar structures with versatile morphology; this includes 2D forms such as nanoplatelets, nanoplates, nanosheets, or nanoflowers. Representative 2D bio‐piezoelectric nanomaterials include black phosphorus,^[^ ^72^ ^]^ boron nitride,^[^ ^73^ ^]^ carbon nitride,^[^ ^74^ ^]^ monolayer transition metal dichalcogenide.^[^ ^75^ ^]^ As a typical in‐plane bio‐piezoelectric material, a monolayer MoS_2_ is constructed from a Mo plane that is sandwiched between two S planes, thereby forming a triangular prism structure with Mo atoms in the center, as seen in Figure 2g.^[^ ^75^, ^76^ ^]^ When subjected to an external stress, the Mo^4+^ and S^2−^ are displaced to produce an electric dipole and polarization charges on the surface of the material, thereby providing the material with piezoelectricity.^[^ ^77^ ^]^ In addition, MoS_2_ nanosheets have been extensively studied in biomedicine as a result of its photosensitivity, thermosensitive properties, good redox activity, and extraordinary biocompatibility.^[^ ^78^ ^]^ All the above properties endow MoS_2_ nanosheets with significant potential in biosensing and bioelectronic applications.^[^ ^77^, ^79^ ^]^

## 3 Synthesis and Modification Strategies

To perform targeted biomedical functions, bio‐piezoelectric materials are usually manufactured into two types of platforms, namely thin films and nanoplatforms. Bio‐piezoelectric films with high flexibility can be applied to skin, muscle, and other tissue surfaces for biosensing or disease treatment. More importantly, the large surface area of a bio‐piezoelectric film provides abundant bonding sites for electronic devices (e.g., capacitors, inductors, and resistors) that allows the development of miniaturized or portable biomedical devices. The main synthetic methods of forming bio‐piezoelectric films include magnetron sputtering, pulsed laser deposition, and solution casting. Biopiezoelectric nanoplatforms exhibit many unique advantages,^[^ ^77^ ^]^ such as extremely small size, high biocompatibility, large specific surface area, and excellent piezoelectric performance, which greatly expand their application prospects in biomedicine. With the increase of research effort on the synthesis and modification methods, bio‐piezoelectric nanoplatforms for the field of biomedicine have become a topic of intense research interest.^[^ ^2^ ^]^ To prepare bio‐piezoelectric nanoplatforms, versatile strategies have been developed in recent years, including mechanical exfoliation, chemical exfoliation, vapor phase deposition, hydrothermal, and sol‐gel method.

### 3.1 Bio‐Piezoelectric Thin Films

With the emergence of micro‐electric‐mechanical systems in biomedicine applications, bio‐piezoelectric thin films have become an ideal platform for the efficient integration of multiple components at small scales. Bio‐piezoelectric thin films exhibit good mechanical flexibility, easy production, low cost, and high stability.^[^ ^80^ ^]^ Moreover, bio‐piezoelectric thin films can be readily combined with semiconductor materials, thus realizing a sensitive response to micro‐mechanical pressures.^[^ ^17^, ^80^ ^]^ To date, a range of approaches such as magnetron sputtering, pulsed laser deposition, and solution casting have been investigated to prepare high‐quality bio‐piezoelectric thin films.

**Figure 3:**
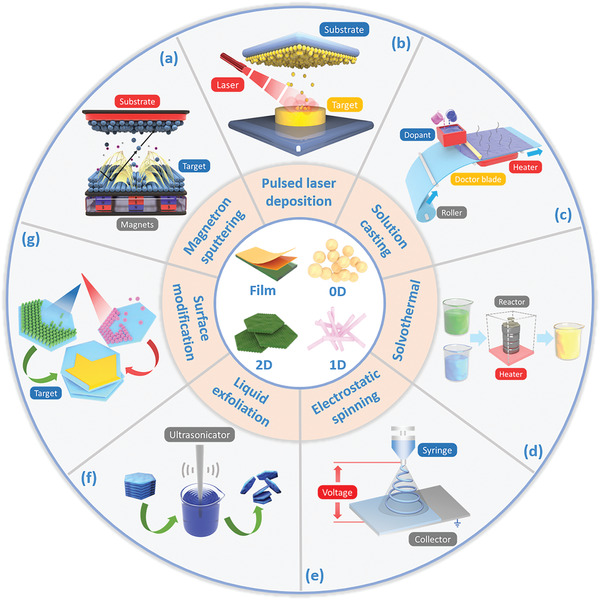
Schematic of the synthesis and modification strategies of bio‐piezoelectric platforms.

Magnetron sputtering is a relatively mature method for thin film preparation, which can be used to prepare films of various substrates including metals, semiconductors, ceramics, and polymers.^[^ ^81^ ^]^ It possesses the merits of fast film formation, high film density, and good film formation consistency.^[^ ^81a^ ^]^ During magnetron sputtering, electrons are accelerated by an electric field between a target and substrate, and are simultaneously bound by a magnetic field. As a result, electrons are able to collide with gas molecules, thereby increasing the ionization rate of plasma.^[^ ^82^ ^]^ Under the action of a high‐voltage electric field, the plasma collides with the target to release target atoms, which subsequently travel to the substrate and form a thin film, as seen in **Figure** **3a**.^[^ ^25^, ^81^ ^]^ Magnetron sputtering can produce thin films with tailored piezoelectricity and conductivity via controlling deposition conditions, such as gas flow rate, substrate temperature, deposition rate, sputtering gas pressure, and annealing conditions. Using polyethylene terephthalate (PET) as a substrate, Costa et al. deposited a series of ZnO bio‐piezoelectric thin films through varying the oxygen flux during a direct current magnetron sputtering process.^[^ ^82a^ ^]^ The obtained ZnO thin film was well adhered to the PET substrate with a uniform film structure and good mechanical strength. At the same time, the surface deposition of ZnO thin film increased the surface energy and hydrophobicity of the PET substrate surface. As a result, the ZnO thin film prepared under an oxygen flux possessed a high piezoelectric coefficient and good adhesion to the substrate.^[^ ^82a^ ^]^

Pulsed laser deposition (PLD) is another method to produce bio‐piezoelectric thin films at low temperature with a clean interface. High‐energy laser pulses are focused on the target surface in an ultrahigh vacuum system. The target materials are then rapidly vaporized and deposited on the substrate as a thin film, as seen in Figure 3b.^[^ ^25^, ^83^ ^]^ The use of noncontact laser heating effectively avoids sample contamination to provide films of high quality. In this regard, PLD technology has been widely utilized for preparing biomedical microdevices.^[^ ^83^ ^]^ Scarisoreanu et al. used the PLD method to grow high‐quality lead‐free bio‐piezoelectric thin films of (1‐*x*)Ba(Ti_0.8_Zr_0.2_)TiO_3_‐*x*(Ba_0.7_Ca_0.3_)TiO_3_, *x* = 0.45 (BCZT 45) on a Pt/Si substrate, and then deposit it on a Kapton polyimide polymer substrate.^[^ ^84^ ^]^ The obtained film not only exhibited high piezoelectric performance, but it also demonstrated excellent biocompatibility and flexibility due to the use of compliant Kapton substrates. In vitro studies revealed that BCZT 45 coatings on a Kapton polymer substrates can promote the adhesion and osteogenic differentiation of stem cells, demonstrating their application prospects for bone repair. Nevertheless, the need for relatively expensive equipment and the complicated nature of the PLD process can restrict its large‐scale application.

Solution casting is a simple and commonly used process for thin film preparation. In this process, a powder sample is combined with a suitable dispersant to form a uniformly dispersed slurry, and a thin film is prepared on a casting machine (Figure 3c).^[^ ^85^ ^]^ Due to the simplicity of the equipment and an ability to achieve a continuous and automated operation with a high production yield, the solution casting method has been widely employed for the preparation of bio‐piezoelectric films. Hosseini et al. synthesized a freestanding bio‐piezoelectric film from glycine and chitosan via the solution casting process.^[^ ^86^ ^]^ For this film, β‐glycine crystals were crystallographically oriented within a chitosan matrix, providing the film with high biocompatibility and flexibility, which can be processed into bio‐piezoelectric pressure sensors for wearable devices.

As a whole, bio‐piezoelectric thin films as biomedical platforms present unique advantages, including high mechanical flexibility, lightweight nature, and excellent sensitivity to surface micropressure. However, the 2D nature of micro‐meter thin‐films limits their broader biomedical applications, especially for those that require 3D structures (e.g., scaffolds). Moreover, the small micro‐meter sizes restrict their biomedical performance, such as the inability to induce or stimulate cells at the nanoscale.

### 3.2 Bio‐Piezoelectric Nanoplatforms

Despite the great potential of bio‐piezoelectric nanoplatforms in biomedicine, it remains a significant challenge to prepare high quality bio‐piezoelectric nanoplatforms with well‐defined morphologies (e.g., nanoparticles, nanofibers, nanowires, and nanoplatelet) and controlled crystalline phases (e.g., cubic, quadrangle, and polyphase). A variety of methods have been developed to fabricate high‐quality bio‐piezoelectric nanoplatforms, such as solvothermal, hydrothermal, electrostatic spinning, and mechanical exfoliation.

Solvothermal and hydrothermal synthesis are efficient methods for the controlled synthesis of bio‐piezoelectric nanoplatforms with a variety of morphologies. During their preparation, chemical reactions are undertaken in a sealed autoclave at high temperature, thereby producing high quality nanocrystals via one‐pot reactions, as shown in Figure 3d.^[^ ^87^ ^]^ Wang et al. prepared ferroelectric tetragonal BaTiO_3_ nanoparticles with an average size of 130 nm through hydrothermal reactions between Ti(C_4_H_9_O)_4_ and Ba(OH)_2_.^[^ ^27c^ ^]^ The BaTiO_3_ nanoparticles provided high piezoelectric activity for the generation of radicals (·OH or ·O_2_^−^) during the application of ultrasonic vibrations to facilitate the cleaning of teeth. The hydrothermal method with its simple operation, low cost, low reaction temperature, and wide applicability for producing various morphologies, sizes, and dimensions can also produce hierarchical nanostructures with specific geometry.^[^ ^87^ ^]^ Ha et al. reported on the formation of ZnO nanowire‐decorated polydimethylsiloxane micropillar arrays via a hydrothermal process, where the ZnO nanowires were grown on polydimethylsiloxane micropillars with a high aspect ratio and precisely controlled dimensions.^[^ ^88^ ^]^ The obtained ZnO nanowire array demonstrated a high degree of bending, ultrafast response, and low thermal expansion, and could be fabricated as a flexible electronic skin.

Electrostatic spinning is a common method for nanofiber preparation.^[^ ^1^ ^]^ In brief, the polymer solution is continuously sprayed from a jet, and stretched under a high electric field to form electrospinning nanofibers on a receiving device, as shown in Figure 3e.^[^ ^89^ ^]^ The formed fiber membranes exhibit good elasticity and high tolerance to applied strains.^[^ ^1^ ^]^ Specifically, the use of a high electric field promotes material polarization during the spinning process, providing the nanofibers with excellent piezoelectric properties.^[^ ^18a^ ^]^ Bairagi and Ali developed KNN/ZnO incorporated PVDF nanocomposites via electrospinning.^[^ ^90^ ^]^ During the electrospinning process, the PVDF polymer underwent mechanical stretching and in situ poling, which transformed the nonpolar α‐phase into a highly polar β‐phase to enhance its piezoelectric properties. Bio‐piezoelectric nanofiber networks can be prepared with a similar morphology to natural tissue by regulating the composition of the precursor solution and electrospinning parameters, such as flow rate, voltage, concentration, and the distance between needle tip and receiver. For example, Jacob et al. prepared bio‐piezoelectric nanofiber scaffolds from poly‐3‐hydroxybutyrate‐3‐hydroxy valerate and BaTiO_3_ nanoparticles, where both the morphology and pore size were similar to natural cartilage by optimizing the spinning parameters.^[^ ^91^ ^]^ The addition of BaTiO_3_ nanoparticles not only enhanced the mechanical properties and piezoelectric coefficients of the poly‐3‐hydroxybutyrate‐3‐hydroxy valerate, but also prolonged its degradation time. Accordingly, the obtained scaffolds exhibited excellent mechanical properties and piezoelectric coefficients, which were comparable to natural cartilage.

Monolayer nanomaterials can be exfoliated from their bulk counterparts under certain mechanical forces, which breaks the weak van der Waals’ force between layers. This method is termed mechanical exfoliation, and is also known as the “Scotch tape method.”^[^ ^77^ ^]^ As a physical separation process, the exfoliation process is simple and rapid for the preparation of bio‐piezoelectric nanomaterials. However, mechanical exfoliation also possesses several inherent shortcomings, such as inhomogeneity of products, low efficiency of the stripping operation, and poor control of nanomaterial morphology. Recently, liquid exfoliation has been developed as an extension of mechanical exfoliation. In this case, the weak van der Waals interactions between adjacent layers of bulk crystals are broken by ultrasonic treatment in an appropriate solvent or surfactant, see Figure 3f.^[^ ^77^, ^92^ ^]^ The solvent molecules can prevent the re‐stacking and aggregation of the lamellar products by forming a protecting layer on their surface.^[^ ^93^ ^]^ For example, Wu et al. exfoliated monolayer MoS_2_ nanosheets from a bulk MoS_2_ powder in *N*‐methyl‐2‐pyrrolidone by ultrasonic‐assisted liquid‐phase exfoliated method.^[^ ^94^ ^]^ Our group has prepared free‐standing black phosphorus nanosheets via liquid exfoliation of black phosphorus crystals in *N*‐methyl‐2‐pyrrolidone solvent.^[^ ^95^ ^]^

### 3.3 Modification and Engineering Methods

To meet the critical requirements of biomedical applications, ideal bio‐piezoelectric platforms should possess not only excellent piezoelectric performance, but they should also exhibit suitable surface properties that are tailored to their application; these can include hydrophilicity, roughness, and porosity. Moreover, bio‐piezoelectric platforms are expected to be biocompatible and biodegradable to ensure biosafety and should exhibit sufficient mechanical properties to provide long‐term flexibly and durability. However, many piezoelectric materials cannot fulfill all of the above‐mentioned requirements. In this regard, surface modification and engineering of piezoelectric materials have become particularly important to achieve the desired biomedical performance and combination of properties. (Figure 3g).

To improve piezoelectric properties of bio‐piezoelectric polymers, the integration of nanofillers such as BaTiO_3_, ZnO, metal nanoparticles, graphene oxide, and carbon nanotubes into a polymer matrix has been demonstrated to be an effective method.^[^ ^20^ ^]^ For example, the addition of nanofillers into PVDF generates an electrostatic interaction with the surrounding PVDF chains and influences the chain orientations, thereby improving the overall piezoelectric response of the composites.^[^ ^18^, ^41^ ^]^ Similarly, Deng et al. improved the piezoelectric performance of PVDF film by wrapping ZnO nanospheres into PVDF electrospinning nanofibers.^[^ ^96^ ^]^ The ZnO nanospheres not only enhanced the local electric field during the electrospinning process to increase the fraction of β‐phase PVDF crystals, but also provided a synergistic effect with PVDF nanofibers to promote the piezoelectric performance of the composites. In addition, the piezoelectric properties of the composites could be readily controlled by adjusting the weight ratio between the ZnO nanospheres and PVDF polymer.

To improve piezoelectric performance of inorganic bio‐piezoelectric materials, the crystal grain size can be tuned to adjust the piezoelectric properties. For example, BaTiO_3_ ceramics possess good piezoelectric performance (maximum value of 519 pC N^–1^) and high dielectric constant (maximum of 6079) when their grain size is ≈1 µm.^[^ ^97^ ^]^ Moreover, the piezoelectric properties of bio‐piezoelectric nanomaterials can be adjusted by altering their morphology since surface area changes the long‐ and short‐range ordering of dipoles.^[^ ^98^ ^]^ Shirazi et al. improved the piezoelectric coefficient of BaTiO_3_ nanofibers by decreasing the fiber diameter.^[^ ^98^ ^]^ In addition, since the mechanical properties are highly related to geometry,^[^ ^99^ ^]^ the creation of well‐defined and optimized morphologies can have a positive effect on overall piezoelectric response. For example, Zhang et al. prepared a piezoelectric energy generator based on PZT microcubes and P(VDF‐TrFE) by a solution casting method.^[^ ^100^ ^]^ Since the cube‐shaped particles possessed pronounced stress concentrators at the particle corners and edges, they exhibited superior piezoelectric activity compared to a generator based on spherical PZT particles.

Chemical vapor deposition (CVD) is a conventional method to prepare bio‐piezoelectric nanomaterials with high purity, good crystallinity, facile controllability, and tunable thickness. Based on these merits, CVD has been widely employed to synthesize monolayer transition metal dichalcogenide,^[^ ^66^, ^101^ ^]^ such as MoS_2_ monolayers.^[^ ^102^ ^]^ However, a large number of intrinsic point defects, such as sulfur (S) vacancies, can be formed during the CVD process, which limits the piezoelectric properties of a monolayer transition metal dichalcogenide. To overcome this shortcoming, Han et al. prepared a S‐vacancy passivated MoS_2_ nanosheet based on CVD growth through a S treatment.^[^ ^102c^ ^]^ Since S vacancies on the MoS_2_ surface tend to covalently bond with the S functional group, the S atom can form a chemical bond with the S vacancy by capturing free electron, thus passivating the S vacancy. Compared with untreated MoS_2_, the S vacancy passivated MoS_2_ nanosheets present significantly enhanced piezoelectric performance, which is considered to be a potential power source for wearable electronic devices.

The surface structure of bio‐piezoelectric materials is a crucial parameter for biomedical applications. For instance, in tissue regeneration, the wettability of the material surface can regulate cell adhesion and cell spreading. The presence of a suitable level of porosity supports blood circulation and the movement of biologically active substances. The surface roughness of the material can also promote cell adhesion, proliferation, and differentiation.^[^ ^103^ ^]^ For example, Qian et al. prepared a ZnO‐loaded polycaprolactone (PCL) piezoelectric nanogenerator scaffold by 3D injectable multilayer biofabrication technology.^[^ ^104^ ^]^ The distribution of ZnO nanoparticles increased the surface roughness of the scaffold and induced a polarization of its surface, which was beneficial to both cell adhesion and proliferation. The high hydrophobicity of the piezoelectric PVDF polymer hinders cell attachment and expansion, thereby limiting its application in the field of tissue engineering. Kitsara et al. prepared PVDF nanofiber scaffolds with a super‐hydrophilic surface through a plasma treatment, which stimulated the adhesion and diffusion of osteoblasts without the need for an external power supply.^[^ ^105^ ^]^ Post‐treatment with an oxygen plasma altered the surface chemical composition of the PVDF scaffold, which led to long‐term and stable hydrophilicity.

Biocompatibility and biosafety are the primary factors that determine the successful application of a bio‐piezoelectric material in biomedicine. As an example, PZT possesses a high piezoelectric charge constant and excellent electromechanical properties, making it attractive for constructing biological microelectronic devices.^[^ ^100^, ^106^ ^]^ However, the toxicity of PZT as a result of the presence of lead (Pb) reduces its biocompatibility when used in conjunction with living cells. Therefore, effort has been devoted to improving the biocompatibility of PZT via surface modification and engineering. The use of coatings to provide biocompatible layers is currently a widely employed strategy to improve the biocompatibility and biosafety of PZT.^[^ ^107^ ^]^ Sakai et al. treated the PZT surface with titanium to enhance its biocompatibility.^[^ ^108^ ^]^ Recently, Kim et al. encapsulated Mn doped (1‐*x*)Pb(Mg_1/3_Nb_2/3_)O_3_‐(*x*)Pb(Zr,Ti)O_3_ film with biocompatible passivation epoxy to minimize the overall cytotoxicity and inflammatory reactions.^[^ ^109^ ^]^ In contrast, developing new piezoelectric materials with both high piezoelectric activity and biocompatibility is another effective strategy to overcome the toxicity issue of PZT. For example, Yuan et al.^[^ ^15a^ ^]^ developed a biocompatible piezoelectric nanogenerator using (1‐*x*)Ba(Zr_0.2_Ti_0.8_)O_3_‐*x*(Ba_0.7_Ca_0.3_)TiO_3_ (BZT‐BCT) nanowires. BZT‐BCT was measured with a comparable piezoelectric coefficient (≈620 pC N^‐1^) to that of many conventional PZTs (200–710 pC N^‐1^),^[^ ^15^, ^110^ ^]^ but with superior biocompatibility and biosafety.

Biodegradability is another important requirement for bio‐piezoelectric platforms. For bio‐piezoelectric film‐based implants, their degradation rates should be sufficiently slow to ensure stable and long‐term performance. For bio‐piezoelectric nanoplatforms, their biodegradability and excretion are examined to avoid the accumulation of material in the body which can have a potentially adverse effect on tissues. To control material biodegradability, Huang et al. deposited an ultra‐thin atomic layer film of alumina to tune the degradation rate of electronic devices in water.^[^ ^111^ ^]^ The biodegradation rate of the electronic device was linearly dependent on the thickness of the alumina coating layer, thus allowing precise control over the lifetime of implantable devices.

## 4 Biomedical Applications of Bio‐Piezoelectric Platforms

The electromechanical conversion characteristics of bio‐piezoelectric platforms enable them to convert external stimuli (ultrasonic waves, pressure, motion) into electrical energy, which not only overcomes the limitation of battery life but can efficiently sense any environmental changes for real‐time biosensing. Moreover, bio‐piezoelectric platforms that use polarized materials with an internal electrical field can regulate a variety of physiological behaviors of cells, such as growth, migration, differentiation, and apoptosis, thereby exhibiting therapeutic effects on diseases. Overall, the biomedical applications of bio‐piezoelectric platforms can be divided into two major groups: biosensing and disease treatment.

### 4.1 Biosensing

The concept of a biosensor was first proposed by Professor Leland C. Clark Jr in 1962.^[^ ^112^ ^]^ Over the past decade, biosensors have gone through rapid development with the trend of being miniature, wearable, portable, and appropriate for potential commercialization.^[^ ^113^ ^]^

Conventional capacitor‐ and inductor‐based biosensors involve the detection of a change in dielectric properties according to the characteristic bonding of the electrode surface.^[^ ^114^ ^]^ However, due to the need for an external power supply, the practical applications of such biosensors can be restricted by their large volume, low structural flexibility, short battery life, and poor biocompatibility.^[^ ^115^ ^]^ Nevertheless, bio‐piezoelectric biosensors have attracted research interest because of their ease of operation, flexible structure, biocompatibility, low detection limit, high sensitivity, and accuracy.^[^ ^3^, ^115^, ^116^ ^]^ More importantly, these sensors are capable of non‐contact sensing and self‐powered supply under certain conditions, which opens a new era for biosensors.^[^ ^117^ ^]^

#### 4.1.1 Monitoring of Physical Health

To realize real‐time health monitoring, traditional implantable medical electronics (IMEs) are driven by an external power supply (e.g., batteries) which often have a poor structural flexibility.^[^ ^114^ ^]^ Batteries have to be surgically replaced when expired, leading to an increased infection risk to patients.^[^ ^23^, ^114^ ^]^ Due to the development of bio‐piezoelectric materials, a variety of miniature and intelligent bio‐piezoelectric biosensors have been developed for detecting a variety of physiological signals. In contrast to traditional IMEs, bio‐piezoelectric biosensors are able to harvest mechanical energy from body motion and convert it into electricity to achieve self‐powered operation and a degree of autonomy, thus avoiding the problem of short battery life in traditional IMEs.^[^ ^17^, ^19^ ^]^ In addition, polymer‐based piezoelectric biosensors with extraordinary flexibility are responsive to small levels of deformation and forces, and are suitable to sensitively monitor physiological signals in the body.^[^ ^118^ ^]^

**Figure 4:**
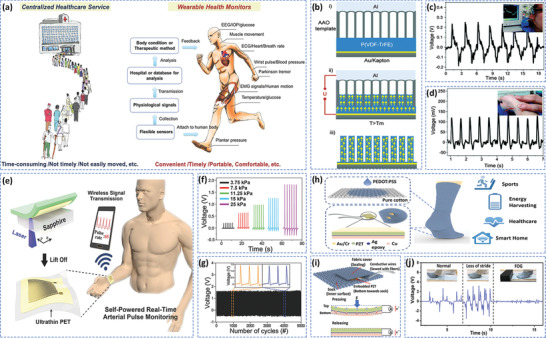
a) Wearable and attachable health monitoring devices provide a convenient way for portable and decentralized healthcare. Reproduced with permission.^[^ ^119^ ^]^ Copyright 2017, Wiley‐VCH. b) Construction of P(VDF‐TrFE) nanowires, and their application in detecting c) respiration and d) pulse. Reproduced with permission.^[^ ^32^ ^]^ Copyright 2015, Royal Society of Chemistry. e) Schematic of fabrication process for self‐powered pressure sensor. f) Generated output voltage of the piezoelectric pressure sensor under different pressure conditions, and g) mechanical durability test of the piezoelectric sensor. Under repeated pushing/release conditions with a frequency of 2 Hz and pressure of 20 kPa. Insets show a partially magnified output voltage versus time. Reproduced with permission.^[^ ^122^ ^]^ Copyright 2017, Wiley‐VCH. h) Schematic of PEDOT:PSS coated triboelectric sock integrated with PZT force sensors. i) Working principle of PZT chip and integration strategy in the sock. j) Mimetic walking pattern of Parkinson's disease patient under three conditions: normal, loss of stride, and freezing of gait (FOG). Reproduced with permission.^[^ ^124^ ^]^ Copyright 2019, American Chemical Society.

Recently, wearable and attachable health monitoring platforms have been proposed that are based on flexible electronic devices, as seen in **Figure** **4a**.^[^ ^119^ ^]^ This form of physiological signal monitoring platform is portable and easily attached to skin or tissues, thus circumventing the need for painful surgery for implantation. Chen et al. constructed a self‐powered biosensor based on a bio‐piezoelectric nanowire array with a vertical arrangement and a preferential polarization orientation for monitoring vital signs.^[^ ^32^ ^]^ A voltage was applied to an electrode pair that was composed of a nano‐porous aluminum oxide template and a conductive substrate coated with P(VDF‐TrFE) bio‐piezoelectric film. The electrical growth and polarization of the P(VDF‐TrFE) nanowires were carried out in the nanopores. The confinement effect of the nanometer template promoted the alignment of polymer dipoles along the vertical direction of the nanowires, as shown in Figure 4b. The application of external mechanical forces led to the nanowires to produce detectable piezoelectric signals. The biosensor exhibited a high sensitivity and flexibility, which was suitable for detecting subtle pressure changes from human activities, including human respiration and pulse rate; this is shown in Figure 4c,d. In addition, Liu et al. created a wearable self‐powered biosensor based on a flexible PVDF nanogenerator, which was used to monitor human respiration and healthcare in real time.^[^ ^120^ ^]^ The PVDF membrane, prepared by electrospinning, was used as the bio‐piezoelectric layer because of its high piezoelectric voltage constant. During breathing, the flexible PVDF membrane on the wearable device was compressed or stretched to produce continuous electrical signals for monitoring respiration in real time. Compared with organic bio‐piezoelectric materials, inorganic bio‐piezoelectric materials, such as BaTiO_3_ and PZT, usually exhibit superior piezoelectric properties,^[^ ^121^ ^]^ thereby producing a higher piezoelectric output for self‐powered biosensors. As a result, Park et al. designed a self‐powered flexible piezoelectric biosensor based on a PZT thin film for real‐time arterial pulse monitoring, see Figure 4e.^[^ ^122^ ^]^ The piezoelectric PZT thin film was transferred onto an ultrathin plastic substrate, and then conformally attached to a human wrist using a biocompatible liquid bandage spray. The biosensor exhibited high mechanical flexibility and could be stably deformed in response to blood vessel movements, and the generated electrical signals could be transmitted wirelessly to a smartphone for real‐time monitoring of an arterial pulse. The biosensor showed not only an accurate piezoelectric response (Figure 4f), but also exhibited an excellent mechanical stability after 5000 cycles (Figure 4g).

Wearable electronic textiles based on the piezoelectric effect have attracted attention due to their lightweight nature and high flexibility. Such textiles can also be used in self‐powered wireless sensors to monitor human health.^[^ ^123^ ^]^ Unlike biosensors driven by solar or thermal energy, wearable electronic textiles can harvest energy from human movement.^[^ ^20^ ^]^ Since human activities often rely on mechanical motion, harvesting energy from body motion in daily activities is of significant interest for wearable electronic devices.^[^ ^20^ ^]^ Zhu et al. developed a self‐powered and self‐functional sock for sensing and monitoring physiological signals such as gait and contact force, see Figure 4h.^[^ ^124^ ^]^ The system incorporated a poly(3,4‐ethylenedioxythiophene) polystyrene sulfonate (PEDOT:PSS) coated fabric triboelectric nanogenerator and a PZT piezoelectric chip, as seen in Figure 4i. The sock transmitted the characteristic waveforms as the wearer walked, providing an easy approach to recognize walking patterns for healthcare applications, in particular for gait monitoring of patients with Parkinson's disease, as shown in Figure 4j. Mao et al. developed a self‐powered piezoelectric‐biosensing textile based on tetrapod‐shaped ZnO (T‐ZnO) nanowires for physiological monitoring of individual sports.^[^ ^125^ ^]^ The T‐ZnO nanowires were firmly anchored on the textiles using a PVDF binder to harvest the mechanical energy from motion and output electrical signals. These fabrics could be worn on different areas of the skin to monitor a variety of physiological states. Such piezoelectric biosensors are suitable for continuous monitoring and sensing, and ultimately stimulate the development of self‐powered wearable devices.

#### 4.1.2 Disease Diagnosis

**Figure 5:**
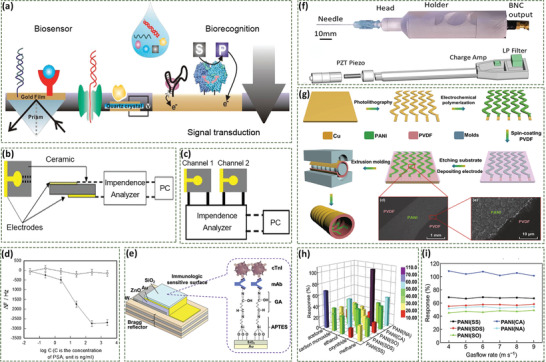
a) Biosensors combine biorecognition and signal transduction elements for quantitative or semiquantitative analysis of biologically relevant analytes. Reproduced with permission.^[^ ^126^ ^]^ Copyright 2017, American Chemical Society. Schematic of b) ceramic resonator and c) dual ceramic resonators in biosensors. d) Frequency changes with PSA concentration. Reproduced with permission.^[^ ^106^ ^]^ Copyright 2013, Elsevier. e) Biosensor for detection of cardiac biomarkers. Reproduced with permission.^[^ ^131^ ^]^ Copyright 2020, Elsevier. f) Schematic of the piezoelectric needle sensor. Reproduced with permission.^[^ ^132^ ^]^ Copyright 2019, Springer Nature. g) Fabrication process of the self‐powered breath analyzer, and SEM images of PANI/PVDF interface. h) Response of five sensing units to 600 ppm gases, and i) relationship between responses and gas flow rates. Reproduced with permission.^[^ ^134^ ^]^ Copyright 2018, Springer Nature.

Biosensors integrating recognition and signal transduction have been widely used in the quantitative or semiquantitative analysis of biologically relevant analytes, see **Figure** **5a**.^[^ ^126^ ^]^ The biosensing process is generally based on changes at an interface after the combination, or reaction, between analytes and recognition elements on the transducer surface.^[^ ^127^ ^]^ In this strategy, oscillating piezoelectric resonators as signal transducers are coated with recognition molecules, such as antibodies or enzymes. The changes in the fundamental frequency of the piezoelectric resonators are recorded to provide quantitative or semiquantitative analysis of target molecules.^[^ ^128^ ^]^ Currently, high‐quality quartz crystals are widely used as piezoelectric transducers, whereas their high cost and moderate sensitivity limit their application in piezoelectric sensors.^[^ ^129^ ^]^ Su et al. developed a biosensor using a PZT ceramic resonator as a transducer in order to detect cancer biomarkers for the preliminary diagnosis of cancer, as shown in Figure 5b,c.^[^ ^106^ ^]^ The prostate‐specific antigen (PSA) antibody or α‐fetoprotein antibody was modified on the surface of the resonator. The relative frequency of the resonator was changed by the antibody‐antigen interaction, thereby realizing quantitative detection of PSA and α‐fetoprotein in serum. Compared with traditional quartz crystals, the ceramic resonator was more sensitive and cost‐effective, see Figure 5d, providing the possibility for creating sensor arrays for multiplex detection in the future.

In addition to cancer, piezoelectric biosensors have been also employed to detect biomarkers of many other diseases. Pohanka et al. developed a piezoelectric immunosensor for detection of inflammatory marker C‐reactive protein in blood, providing reference for distinguishing bacterial and viral infections.^[^ ^130^ ^]^ Liu et al. constructed a biosensor based on a ZnO bio‐piezoelectric film for detecting a biomarker (cardiac troponin I, cTnI) of acute myocardial infarction, Figure 5e.^[^ ^131^ ^]^ The formation of micron‐thick bio‐piezoelectric films allowed the resonator to operate at high frequency, up to several thousand megahertz, thus achieving a detection limit as low as a single molecule.

Beyond biomarker detections, piezoelectric needle sensors have been developed to diagnose pathological tissues based on their mechanical heterogeneity and density, see Figure 5f.^[^ ^132^ ^]^ When a piezoelectric needle is inserted into a tissue, the electric current signal is converted into an electrical signal by a piezoelectric sensor to directly examine pathological changes in tissue. Such a label‐free strategy simplifies the screening methods for the diagnosis of malignant thyroid nodules, solid tumors, and other diseases.

In most cases, disease diagnosis with biosensors usually involves painful and invasive sampling procedures, such as the drawing of blood or cerebrospinal fluid collection. As a result, a noninvasive and convenient method is preferable for disease diagnosis at the early stage or continuous testing.^[^ ^133^ ^]^ Fu et al. developed a self‐powered breath analyzer based on a polyaniline/polyvinylidene fluoride (PANI/PVDF) piezoelectric gas‐sensing array, see Figure 5g.^[^ ^134^ ^]^ The PVDF film in the device converted the energy associated with exhalation into an electrical signal to achieve a self‐powered supply, and five PANI electrodes with different dopants were employed as the gas‐sensing material. Each sensing unit has a favorable selectivity to a particular gas marker, which leads to the change of the output electrical signals of the corresponding unit; as shown in Figure 5h.^[^ ^135^ ^]^ The biosensor presented constant response/recovery circles under different gas flow rates (Figure 5i). Such a piezoelectric biosensor possesses application prospects for the early diagnosis of a variety of diseases, such as airway inflammation, asthma, liver cirrhosis, and diabetes.

#### 4.1.3 Smart Devices and Bionic Prostheses

**Figure 6:**
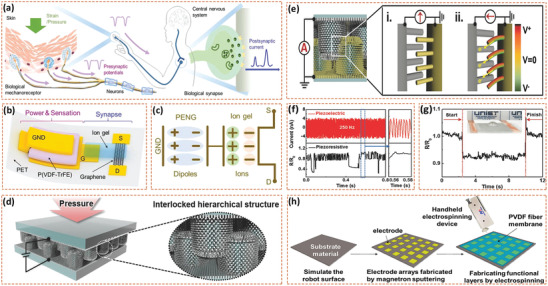
a) Biological sensory nerve system. Mechano‐receptors in human skin receive mechanical stimulus and convert it into the presynaptic potentials. Presynaptic potentials are transmitted to central nervous system through neurons and synapses. b) Schematic of piezotronic graphene artificial sensory synapse and c) coupling effect of piezoelectric potential through an ion gel. Reproduced with permission.^[^ ^136^ ^]^ Copyright 2019, Wiley‐VCH. d) Schematic of piezoresistive e‐skin device based on hierarchical ZnO nanowire arrays. e) Schematic of piezoelectric potential between the interlocked ZnO nanowires (state i). Applied pressure bends the bare ZnO nanowires and creates a piezoelectric potential (state ii). f) High‐frequency vibration sensing capability of piezoelectric e‐skins, and g) minimum detection capability of e‐skins showing the detection of a small water droplet (0.58 Pa) on the e‐skin. Reproduced with permission.^[^ ^88^ ^]^ Copyright 2015, Wiley‐VCH. h) Fabrication of single‐electrode e‐skin. Reproduced with permission.^[^ ^138^ ^]^ Copyright 2018, American Chemical Society.

For smart devices and bionic prostheses, sensory feedback is highly dependent on the performance of the biosensor, which requires maximum simplification of information processing. The piezopotential of piezoelectric devices triggered by mechanical stimuli can be readily captured and analyzed, which can replace traditional sensing components. Chen et al. developed an artificial sensory synapse composed of a piezoelectric nanogenerator and an ion‐gel gated transistor, see **Figure** **6a**–c.^[^ ^136^ ^]^ The piezoelectric properties of the piezoelectric nanogenerator enabled the synaptic device to be self‐powered; while the coupling effect of the piezoelectric potential converts strain information into a postsynaptic current to simulate synaptic functions. Thus, such piezotronic artificial synapses achieves self‐powered sensing and efficient signal processing of external stimuli.

Among the range of haptic feedback devices, electronic skins (e‐skins) have the advantages of high spatial resolution, ultrahigh sensitivity, ultrafast response speed, and excellent durability; these have therefore become a worldwide research focus.^[^ ^22^, ^137^ ^]^ Ha et al. proposed a bioinspired e‐skin composed of hierarchical ZnO nanowire arrays with an interlocked geometry arrangement, as seen in Figure 6d.^[^ ^88^ ^]^ The interlocked ZnO nanowires with piezoresistive (change in resistance with the stress) and piezoelectric sensing capabilities were responsive to tactile stimuli due to ZnO nanowire bending and the change of internanowire contact area, see Figure 6e. The e‐skin could detect not only static stimuli, such as small vibrations and sound stimulation (Figure 6f), but it could also detect high‐frequency dynamic vibrations at up to 250 Hz, see Figure 6g. Wang et al. reported a flexible e‐skin biosensor based on a single‐electrode piezoelectric nanogenerator for simultaneous tactile and temperature sensing.^[^ ^138^ ^]^ The piezoelectric nanogenerator was composed of an electrode array prepared by magnetron sputtering and an PVDF bio‐piezoelectric layer by electrospinning, see Figure 6h. The electrospinning bio‐piezoelectric PVDF nanofibers provide the biosensor with excellent flexibility and self‐powered capability for future applications as a bionic e‐skin.

In addition to tactile sensing, self‐powered wearable e‐skins for sensing taste have been developed.^[^ ^139^ ^]^ A gustation (tasting) e‐skin was formed which was based on an enzyme‐modified ZnO nanowire array on a flexible substrate of patterned electrodes, which can operate in a liquid solution, and collect data directly in the biological environment, and acts in a similar way as a taste bud on the tongue. The coupling between the piezoelectric‐enzymatic reaction allows the e‐skin to harvest mechanical energy from the body for self‐powered sensing. Different enzymes on the hydrothermally synthesized ZnO nanowire array catalyze specific enzymatic reactions of taste‐producing substances, thereby generating piezoelectric signals for taste sensing.

In addition to tactile sensing, self‐powered wearable e‐skins for sensing taste have also been developed, see Figure 6i.^[^ ^131^ ^]^ A gustation (tasting) e‐skin was formed which was based on an enzyme‐modified ZnO nanowire array on a flexible substrate of patterned electrodes, which can operate in a liquid solution, and collect data directly in the biological environment, and acts in a similar way as a taste bud on the tongue. The coupling between the piezoelectric‐enzymatic reaction allows the e‐skin to harvest mechanical energy of body for self‐powered sensing. Different enzymes on the hydrothermally synthesized ZnO nanowire array catalyze specific enzymatic reactions of taste‐producing substances (Figure 6j), thereby generating piezoelectric signals for taste sensing.

### 4.2 Disease Treatment

Bioelectricity is a cell communication and information transportation mode generated from a variation in membrane potentials across individual or fields of cells. It plays an important role in multiple biological processes,^[^ ^140^ ^]^ including cell activity (e.g., migration, proliferation, differentiation, and intracellular communication), and tissue functions such as neural conduction and tissue reparation.^[^ ^21^ ^]^ Moreover, a range of studies have indicated the significant potential of bioelectric therapy for a variety of diseases, such as cancer, tissue dysfunction, neurodegenerative disorders, and bacterial infection. Due to their unique electromechanical conversion capabilities, bio‐piezoelectric materials have been demonstrated as an ideal platform for bioelectricity generation and bioelectric therapy.

#### 4.2.1 Cancer Treatment

The anticancer activity of bio‐piezoelectric materials is predominantly mediated by the following three processes: piezoelectric stimulation, reactive oxygen species (ROS) generation, and controlled drug delivery.

**Figure 7:**
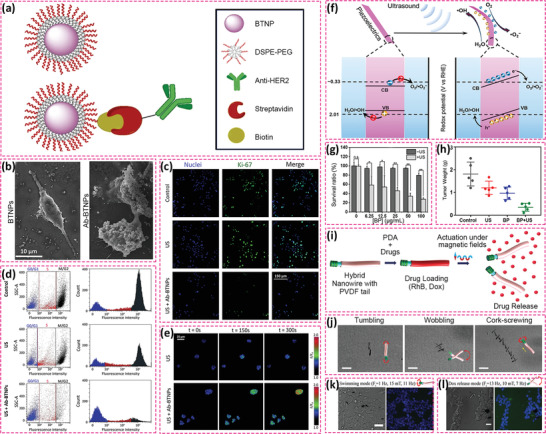
a) Schematic of the functional modification of BTNPs with anti‐HER2 antibody through DSPE‐PEG‐biotin coating. b) SEM images of SK‐BR‐3 cells treated with BTNPs and Ab‐BTNPS. c) Fluorescence images of Ki‐67 expression in ultrasound‐stimulated cells without or with Ab‐BTNPs. d) Flow cytometry assessment of SK‐BR‐3 cell cycle phases (blue stands for G0/G1phases; red stands for S phase and dark gray stands for M/G2 phases). e) Fluorescence images of intracellular Ca^2+^. Reproduced with permission.^[^ ^27a^ ^]^ Copyright 2018, Springer Nature. f) Schematic of piezoelectric polarization for ROS generation under ultrasound irradiation. g) Cell viability of 4T1 cancer cells after the treatment of black phosphorus nanosheets under ultrasound irradiation. h) Average weights of tumors collected on day 14. Reproduced with permission.^[^ ^146^ ^]^ Copyright 2020, American Chemical Society. i) Structure of hybrid nano‐eels for drug delivery. j) Swimming behavior of the hybrid nano‐eels. k) Bright field and fluorescent images of cancer cells with nano‐eels in swimming model, and l) in drug release model. Reproduced with permission.^[^ ^148^ ^]^ Copyright 2019, Wiley‐VCH.

In general, upon the application of an external stress, bio‐piezoelectric platforms generate piezoelectric potential that can interfere with ion channels and inhibit the proliferation of cancer cells. As a typical example, BaTiO_3_ has attracted significant attention in cancer therapy due to its excellent piezoelectric properties and high biocompatibility.^[^ ^141^ ^]^ Marino et al. fabricated an innovative bio‐piezoelectric nanoplatform based on BaTiO_3_ nanoparticles for ultrasound‐activated anticancer treatment.^[^ ^27a^ ^]^ This work considered the overexpression of human epidermal growth factor type 2 receptor (HER2) in breast cancers,^[^ ^142^ ^]^ where the bio‐piezoelectric BaTiO_3_ nanoparticles were functionalized with an anti‐HER2 antibody to target breast cancer cells, see **Figure** **7a**–e. Under ultrasonic stimulation, the BaTiO_3_ nanoparticles generated a piezoelectric potential due to the acoustic stress ^[^ ^143^ ^]^ that inhibited the proliferation of cancer cells by interfering with K^+^ channels and Ca^2+^ homeostasis.^[^ ^144^ ^]^

ROS‐based cancer therapy has been demonstrated as a clean and safe therapeutic modality due to it being free of highly toxic chemotherapeutic drugs or ionizing radiation. Thus, significant effort has been devoted to ROS‐based cancer therapy in recent years, such as photodynamic, chemodynamic, radiodynamic, and sonodynamic therapies. Piezoelectric materials as energy transducers convert mechanical energy into electricity, thereby facilitating the production of highly toxic ROS for tumor eradication. Among the various mechanical strains, ultrasound is the most widely used due to its ease of operation and application, high tissue penetrating depth, and minimal tissue damage. In general, ultrasonic waves apply compressive/tensile stresses to piezoelectric materials and induce the generation of electric fields for the separation of free electrons and holes. The generated electrons and holes act as redox‐active sites to subsequently react with H_2_O and O_2_ in its surroundings to yield highly toxic ROS, such as hydroxyl radical (·OH) and singlet oxygen (^1^O_2_).^[^ ^14^, ^145^ ^]^ Therefore, piezoelectric sonosensitizers usually exhibit a high efficiency in terms of ROS production. Li et al. employed piezoelectric black phosphorus nanosheets as a new class of sensitizers for sonodynamic cancer therapy, see Figure 7f‐h.^[^ ^146^ ^]^ Both in vitro and in vivo experiments demonstrated that such a therapeutic strategy is effective in inhibiting tumor growth. Recently, Zhu et al. utilized H_2_O_2_ to perform hydrophilic treatment of tetragonal BaTiO_3_ nanoparticles and embedded them into an injectable hydrogel which catalyzes ROS generation under ultrasound stimulation, exhibiting a positive effect on killing tumor cells.^[^ ^67b^ ^]^

The construction of a controlled drug delivery system has proven to an efficient way to improve drug bioavailability and minimize adverse effects.^[^ ^147^ ^]^ Piezoelectric carriers can respond to mechanical motion and generate electric polarization, which changes the charge distribution on the carrier surface that can influence drug binding and release profiles.^[^ ^148^ ^]^ Mushtaq et al. proposed multi‐functional piezoelectric nanorobots for delivering anticancer drug doxorubicin (Dox) to cancer cells under the control of an external magnetic field, see Figure 7i‐l.^[^ ^148^ ^]^ A polypyrrole (Ppy) nanowire as the head of the nanorobot was decorated by nickel (Ni) rings for magnetic actuation, and a PVDF‐based copolymer tail with spontaneous electric polarization under strain was employed for drug loading. Under the manipulation of an alternating magnetic field, the magnetic head module (Ni‐Ppy) oscillated and induced an electric polarization in the PVDF tail to release the Dox drug via electrostatic repulsion. Such a strategy avoids premature drug leakage from carriers, and accurately releases drugs at targeted locations on demand.

#### 4.2.2 Tissue Regeneration

**Figure 8:**
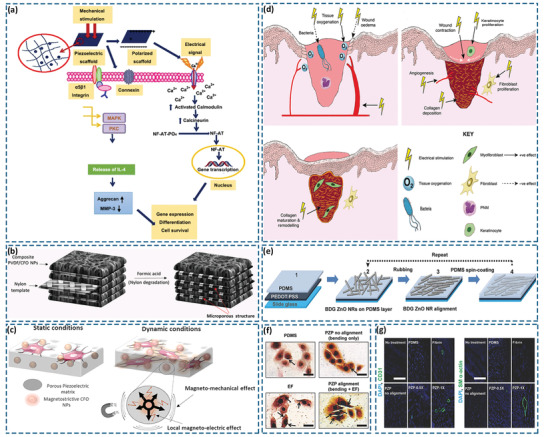
a) Schematic of electrical stimulation to activate Ca^2+^ signal transduction pathway and other transduction pathways. Reproduced with permission.^[^ ^150a^ ^]^ Copyright 2018, Springer Nature. b) Schematic of magnetoactive 3D porous scaffolds. c) Schematic of magneto‐mechanical and local magneto‐electrical properties of 3D scaffolds under the magnetic stimulation. Reproduced with permission.^[^ ^151^ ^]^ Copyright 2019, American Chemical Society. d) Effect of electrical stimulation on the three stages of wound healing. Reproduced with permission.^[^ ^154^ ^]^ Copyright 2016, Wiley‐VCH. e) Fabrication method of the ZnO nanorod‐based piezoelectric dermal patch. f) The von Kossa staining exhibited the condensed calcium deposition (indicated by arrows) in keratinocyte cell membranes after 24 h treatment. g) Immunohistochemical staining of CD31‐positivemicrovessels (green: CD31; blue: nucleus) (left) and SM α‐actin positive arterioles (green: SM α‐actin; blue: nucleus) (right) at the wound healing region 14 d post treatment. Reproduced with permission.^[^ ^153^ ^]^ Copyright 2017, Wiley‐VCH.

When subjected to mechanical stress as a result of body motion or cell migration, a piezoelectric‐based scaffold can generate an electrical potential difference via the piezoelectric effect. In most cases, electrical stimulation can regulate voltage‐gated ion channels, such as calcium channels, to tune the intracellular ion level, thereby promoting cell proliferation and differentiation.^[^ ^149^ ^]^ For example, an increased intracellular Ca^2+^ concentration has been proven to activate calmodulin, calcineurin and calcineurin dephosphorylates nuclear factor, which further translocate into the nucleus to elevate the expression of growth factors, as shown in **Figure** **8a**. In this regard, bio‐piezoelectric materials provide an effective platform for stimulating the regeneration of complex tissues, such as bone and cartilage.^[^ ^149^, ^150^ ^]^ Recently, Fernandes et al. constructed a magneto‐active 3D porous scaffold for bone regeneration which was composed of a bio‐piezoelectric PVDF polymer and magnetostrictive CoFe_2_O_4_ nanoparticles that strain with an applied magnetic field, see Figure 8b.^[^ ^151^ ^]^ The negatively charged CoFe_2_O_4_ NPs interact with the positively charged CH_2_ groups of the bio‐piezoelectric PVDF, thereby inducing the nucleation of the electroactive β‐phase of the polymer and improving magnetoelectric coupling.^[^ ^152^ ^]^ As a result of the magnetic properties of the CoFe_2_O_4_ nanoparticles and the piezoelectric properties of the PVDF polymer, the scaffolds exhibited both magneto‐mechanical and magneto‐electrical effects, both of which synergistically promoted bone tissue reparation under the control of an applied magnetic field, see Figure 8c. In addition, the porous and hydrophilic scaffold structure was beneficial to cell proliferation and penetration within the scaffolds.

Wound repair is also a research focus of tissue regeneration. Current wound‐healing therapies mostly involve a passive healing process, which focuses on reducing wound infection and increasing tissue rehydration at the wound site.^[^ ^153^ ^]^ Compared with passive treatment strategies, bio‐piezoelectric materials can transform motion‐induced mechanical energy into internal electric field to actively accelerate wound healing.^[^ ^21^, ^154^ ^]^ Recent studies have disclosed the specific effects of electrical stimulation on wound healing within three intersecting stages: inflammation, proliferation, and remodeling. In the inflammation stage, electrical stimulation can increase blood flow and tissue oxygenation supply to inhibit bacterial growth and minimize wound edema. In the proliferative phase, electrical stimulation accelerates wound contraction, fibroblast proliferation, angiogenesis, and collagen deposition. In the remodeling stage, electrical stimulation enhances maturation and remodeling of collagens, thereby accelerating wound contraction and increasing wound tensile strength, see Figure 8d.^[^ ^154^, ^155^ ^]^ Therefore, bio‐piezoelectric materials as smart biomaterials generate bioelectric signals under mechanical stimulus to conduct wound repairing functions.^[^ ^156^ ^]^ For example, Bhang et al. developed a hydrothermally synthesized ZnO nanorod‐based piezoelectric dermal patch for skin wound healing. A piezoelectric potential was generated on the piezoelectric dermal patch upon motion‐triggered mechanical deformation, which subsequently induced an electric field to stimulate skin regeneration, see Figure 8e‐g.^[^ ^153^ ^]^

#### 4.2.3 Neurotrauma and Neurodegenerative Treatment

**Figure 9:**
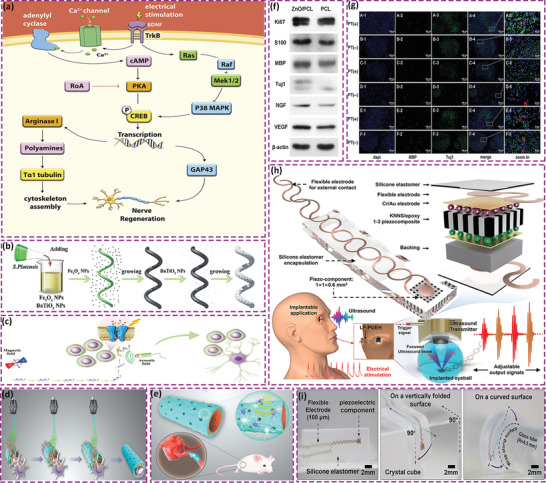
a) Regulation of electrical stimulation on metabolic pathways in neuronal cells. Reproduced with permission.^[^ ^158a^ ^]^ Copyright 2020, Elsevier. b) Schematic of the fabrication of *Spirulina platensis*@Fe_3_O_4_@tBaTiO_3_ micromotor. c) Magnetism‐driven movement to the targeted neural stem‐like cell and ultrasonic‐induced cell differentiation. Reproduced with permission.^[^ ^159^ ^]^ Copyright 2020, Wiley‐VCH. d) Preparation of ZnO loaded PCL conduit via 3D injectable multilayer biofabrication. e) Mechanical stimulation induced piezoelectric effects. f) Western blotting results of Ki67, S100, MBP, Tuj1, NGF, and VEGF in Schwann cells on ZnO/PCL and PCL scaffolds. g) MBP and Tuj1 triple immunostaining image of sciatic nerves acquired from the ZnO/PCL, PCL, and autograft groups: (A1–A5, B1–B5) ZnO/PCL conduit; (C1–C5, D1–D5) PCL conduit; (E1–E5, F1–F5) autograft. PT (+): physical therapy group. PT (‐): nonphysical therapy group. Reproduced with permission.^[^ ^104^ ^]^ Copyright 2020, Wiley‐VCH. h) Schematic of key components of a device which utilizes lead‐free piezoelectric composite as the core component, wavy‐structure‐based flexible electrodes as external contact, and a silicone elastomer as encapsulation. The device provides adjustable electrical outputs driven by ultrasound for electrical stimulation. i) Images of device to demonstrate mechanical properties. Reproduced with permission.^[^ ^161^ ^]^ Copyright 2019, Wiley‐VCH.

A number of nervous system diseases (NSD), such as nerve trauma and neurodegenerative diseases, are a major cause of disability and mortality.^[^ ^157^ ^]^ It has been reported that electrical stimulation upregulates brain‐derived neurotrophic factor (BDNF) and its high‐affinity receptor tropomyosin receptor kinase B (TrkB) in neuronal cells. Via a calcium‐dependent mechanism, up‐regulated BDNF and TrkB increase the expression of regeneration‐associated genes by up‐regulating cyclic adenosine monophosphate (cAMP) pathways, and ultimately promote axon bursting and prevention of growth cone collapse, as shown in **Figure** **9a**.^[^ ^158^ ^]^ Therefore, bio‐piezoelectric materials are able to enhance nerve regeneration via generating electrical stimulation to injured nerves under a mechanical stimulus.

Stem cell‐based therapy is a promising therapeutic modality and has been widely studied for NSD treatment. Liu et al. examined a biodegradable *Spirulina platensis* with Fe_3_O_4_ and BaTiO_3_ nanoparticles formed by electrostatic adsorption, providing a magnetically powered and piezoelectric nanoparticle‐loaded micromotor.^[^ ^159^ ^]^ Under the control of a low‐intensity rotating magnetic field, the micromotor was able to selectively target neural stem cells. Moreover, the piezoelectric properties of the BaTiO_3_ nanoparticles converted ultrasonic energy into a form of electrical stimulation, and activated intracellular Ca^2+^ channels and subsequent signaling cascades to induce neural stem cell differentiation, as shown in Figure 9b,c.

For peripheral nerve regeneration, nerve conduits provide mechanical support and electrical conductivity for the self‐reparation of defected peripheral nerves. In particular, the bioelectrical conductivity of nerve conduits is a critical parameter affecting peripheral nerve viability, axon extension, and signal transmission. Since bio‐piezoelectric nanomaterials generate a piezoelectric potential during the application of an external mechanical load, piezoelectric strategies have been developed to provide an electrically conductive microenvironment to accelerate peripheral nerve reparation. Qian et al. created a bio‐piezoelectric ZnO‐loaded polycaprolactone (ZnO/PCL) composite as a self‐powered nanogenerator scaffold for enhancing motor recovery and neural functions.^[^ ^104^ ^]^ Under the action of an external ultrasonic stimulus, the bio‐piezoelectric ZnO/PCL scaffold showed proangiogenic and proneurogenic effects owing to the mechano‐electric stimulation of Schwann cells. Interestingly, when rats implanted with the bio‐piezoelectric ZnO/PCL scaffold received running practice, their motor recovery was improved due to the creation of bioelectrical milieu for peripheral nerve regeneration; this is shown in Figure 9d–g.

Another therapeutic strategy for NSD is to construct implantable neuroprostheses, such as microelectrodes, which are capable of transmitting information to nerves in the form of electrons, photons, or ions. Implantable devices execute restoring or substituting functions for patients with neurological deficits or disabilities.^[^ ^160^ ^]^ Despite the promise of implantable neuroprostheses, significant challenges remain prior to their successful clinical application. In particular, the long‐term biointegration of the system requires the implant device to have a continuous power supply for stable functionality. Jiang et al. proposed a millimeter‐scale bio‐piezoelectric patch, which was capable of wirelessly self‐powering itself by converting ultrasonic energy into a piezoelectric potential for biological implants.^[^ ^161^ ^]^ The bio‐piezoelectric patch generated tunable electrical output under ultrasonic stimulation, which supplied sufficient power for microdevices and strong current signals for retinal electrical stimulation; this is shown Figure 9h,i.

#### 4.2.4 Antifouling Treatment

**Figure 10:**
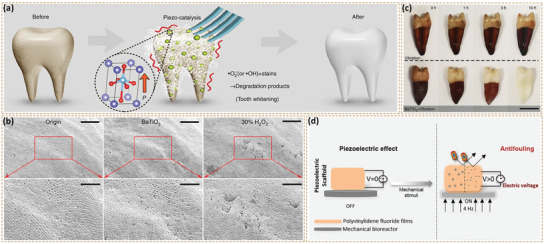
a) Schematic of tooth whitening via piezocatalytic ROS generation. b) SEM images of tooth before (left) and after (middle) piezocatalytic whitening with BaTiO_3_ solution for 3 h, compared with 30% H_2_O_2_ whitening (right) (scale bar = 100 µm). The image below is the magnified area of the marked region (scale bar = 50 µm). c) Photographs of teeth vibrating in pure deionized water (upper) and BaTiO_3_ nanoparticle solution (lower) after 0, 1, 3 and 10 h, respectively (scale bar = 1 cm). Reproduced with permission.^[^ ^27c^ ^]^ Copyright 2020, Springer Nature. d) Schematic diagram of antifouling bio‐piezoelectric scaffold. Reproduced with permission.^[^ ^166^ ^]^ Copyright 2019, American Chemical Society.

Fouling is a complex process in which material from the environment, such as macromolecules, microorganisms, or suspended particles, adhere to the surface reversibly or irreversibly.^[^ ^162^ ^]^ Fouling can cause many health problems, such as bacterial growth, implant rejection, and biosensor insensitivity.^[^ ^162^ ^]^ As a typical example, tooth stains are present in the majority of people, which negatively affects human daily life. To remove tooth stains, dentists currently employ strong oxidants, such as hydrogen peroxide, to bleach teeth and professional equipment to polish teeth. However, these methods usually lead to serious side effects, such as tooth damage, gingival irritation, and acute pulpitis.^[^ ^163^ ^]^ Wang et al. reported on a nondestructive, harmless, and convenient abrasive for teeth whitening based on hydrothermally synthesized bio‐piezoelectric BaTiO_3_ nanoparticles.^[^ ^27c^ ^]^ Under the ultrasonic vibration of an electric toothbrush, the polarized BaTiO_3_ nanoparticles produced ROS (i.e., ·OH and ·O_2_^−^) to whiten pigmented teeth with minimal damage to tooth enamel and surrounding cells, as shown in **Figure** **10a**‐c.

Compared with tooth stains, biological fouling caused by bacteria is a more serious challenge. The adhesion of bacteria on a tissue surface eventually leads to biofilm formation.^[^ ^164^ ^]^ The biofilm not only provides a microenvironment for micro‐organism proliferation and migration, but also protects them from ultraviolet rays and antibacterial agents, which aggravates bacterial resistance against treatment.^[^ ^165^ ^]^ Feng et al. investigated the antifouling properties of bio‐piezoelectric PVDF film from the respect of surface charge variation under mechanical stimuli. The PVDF film was resistant to bacterial adhesion and proliferation under appropriate frequency of mechanical stimuli, as shown in Figure 10d.^[^ ^166^ ^]^

Overall, inorganic bio‐piezoelectric materials usually exhibit higher piezoelectric coefficients compared to bio‐piezoelectric polymers.^[^ ^107^ ^]^ The performance of inorganic bio‐piezoelectric materials in disease treatments is related with their piezoelectric catalytic activity, which is highly dependent on particle size and crystal structure. In this regard, inorganic bio‐piezoelectric nanoplatforms with a nano‐scale size and subjected to nanoconfinement effects to improve activity have been used in biological applications such as drug transportation, cancer killing, tissue repair, and neuromodulation. However, few inorganic bio‐piezoelectric materials are reported with excellent dispersion stability and good biocompatibility, which make current available bio‐piezoelectric nanoplatforms relatively limited. Recent studies have revealed that some biocompatible piezoelectric bulk materials could be readily exfoliated into bio‐piezoelectric nanomaterials, which offers a new strategy to overcome this problem.^[^ ^66^, ^77^ ^]^ Furthermore, material modification and surface engineering can be employed to improve the performance of existing bio‐piezoelectric nanomaterials. In contrast to inorganic piezoelectric materials, bio‐piezoelectric polymers provide good mechanical flexibility, high biological safety, an acoustic impedance similar to human tissue, and ease of processing; these characteristics make them attractive in the field of biomedical electronic devices. Nevertheless, bio‐piezoelectric polymers commonly exhibit moderate piezoelectric properties. To overcome the shortcoming, bio‐piezoelectric composites,^[^ ^26^, ^167^ ^]^ which combine high piezoelectric performance of inorganic piezoelectric materials with flexibility of piezoelectric polymers, have been developed for biomedical applications, including health monitoring, disease diagnosis, and bionic/smart devices. Notably, micro‐ and nanoscale multifunctional piezoelectric biosensors have become the mainstream of bioelectronics. Such a miniatured bio‐piezoelectric device usually requires complex fabrication processes, which can limit its production at mass scale for commercialization. As a result, more effort should be paid to new multifunctional bio‐piezoelectric materials and cost‐effective fabrication technologies.

## 5 Summary and Outlook

As a functional biomedical material, bio‐piezoelectric platforms can provide the merits of low cost, ease of preparation, and stable performance, and have become a research hotspot in the field of biomedicine. The electromechanical characteristics of piezoelectric materials make it possible to transfer the strain from biological movements (such as muscle contraction, body movement, blood circulation, breathing, heartbeats, etc.) into electrical energy. Not only can it be used as an implantable medical device to achieve long‐term and stable energy supply, but it can also be used as a real‐time sensing device to monitor a variety of vital signs such as heart rate, breathing and blood pressure. Piezoelectric biosensors can also lead to the emergence of advanced medical equipment, such as cardiac pacemakers, cochlear implants, artificial retinas, neurostimulators, and electronic skins. While the applications of bio‐piezoelectric platforms have previously focused on bioelectronics, recent advances in bio‐piezoelectric nanomaterials have opened up new opportunities for creating bio‐piezoelectric platforms in biomedicine. Their nanoscale dimensions and ability for electromechanical conversion provide materials with piezoelectric catalytic activity to generate reactive oxygen species for cancer treatment and antifouling. Moreover, bio‐piezoelectric nanomaterials with high piezoelectric coefficients can efficiently respond to small‐scale mechanical strains and thereby regulate biological systems. Their ability for electromechanical conversion allows these fascinating materials to be used as a strong functional platform in a variety of biological applications, such as electronic skins, drug delivery, nerve stimulation, tissue regeneration, wound healing, and cancer treatment.

This review has described the structures and synthesis of bio‐piezoelectric materials, with an emphasis on their modification and design. We have also summarized the latest biomedical applications of bio‐piezoelectric materials, in terms of health monitoring, disease diagnosis, bionic/smart devices, cancer treatment, tissue regeneration, neurotrauma treatment and antifouling. These applications areas, materials, properties, and outcomes are summarized in detail in **Table** **1**.

**Table 1:** Current bio‐piezoelectric platforms and their biomedical applications

Biomedical application	Piezoelectric phase	Morphology	Dimension	Synthesis route	Application	Working site	Working condition	Properties	Ref.
Physical Health Monitoring	P(VDF‐TrFE)	Nanowire	Diameter: ≈400 nm Length: ≈10 µm	Nanotemplate‐based electricity‐grown	Monitor small human activities	Human skin	10 mm × 10 mm device effective area	≈4.8 V maximum voltage, ≈0.11 µA cm^–2^ current density	^[^ ^66^ ^]^
	PZT	Thin film	\	Inorganic‐based laser lift‐off	Monitor epidermal pulse signals	Human skin	Within 30 kPa pressure	0.018 kPa^−1^ sensitivity, 60 ms response time	^[^ ^122^ ^]^
	PVDF	Film	Thickness:≈200 nm	\	Monitor cyclic expand‐contract movement of the chest	Human chest	37 N, 1.4 Hz cyclic mechanical force	≈1.5 V open‐circuit voltage, ≈400 nA short‐circuit current	^[^ ^120^ ^]^
	PZT	Ceramic	Thickness: ≈20 µm	\	Monitor gait signals during movement	Human foot	0.4 MΩ load resistance and 12 N, 1 Hz force	32 µW maximum power per chip (5 mm × 5 mm)	^[^ ^124^ ^]^
	PVDF	Nanofiber	Average diameter: 100–120 nm	Continuous electrospinning	Monitor body motions	Human foot	8.3 kPa of the applied stress amplitude	≈48 V open‐circuit voltage, ≈6 µA short‐circuit current	^[^ ^172^ ^]^
	PVDF BaTiO_3_	Nanofiber	\	Electrospinning	Monitor body motions	Human skin	Within 40 kPa pressure	0.017 kPa^−1^ sensitivity, 290 ms response time	^[^ ^173^ ^]^
	PVDF	Nanofiber	\	Electrospinning	Monitor micropressure changes outside cardiovascular walls	Cardiovascular wall	1.5 Hz, 1 kPa pressure	1154 V cm^–3^ piezoelectric output	^[^ ^115c^ ^]^
	PMN‐PZT	Film	Thickness: ≈20 µm	Solid‐state crystal growth	Monitor the heartbeat	Epicardium	2 cm curvature radius, 0.4 Hz frequency mechanical bending	≈40 V open‐circuit voltage, ≈4.5 µA short‐circuit current	^[^ ^109^ ^]^
Disease Diagnosing	PVDF	Film	\	Spin‐coating	Detection of disease gas markers	Exhaled air	4–9 m s^–1^ airflow rate	0–600 ppm gas markers concentration detection range	^[^ ^134^ ^]^
	ZnO	Film	Thickness: ≈600 nm	Radio frequency sputtering	Detection of acute myocardial infarction markers	Serum	10 µL sample consumption	20 pg mL^–1^ cTnI concentration detection limit	^[^ ^131^ ^]^
	PZT	Ceramic	\	\	Detection of cancer markers	Serum	1 µL sample consumption, within 30 min	0.25 ng mL^–1^ PSA concentration detection limit	^[^ ^106^ ^]^
	PZT	Ceramic	\	\	Detection of mechanical heterogeneity in thyroid tissue lesions	Thyroid tissue	Standard Becton Dickinson (BD) 25 Gauge 3.50″ fine needle	Malignant tissue lesions were rapidly detected based on heterogeneity of tissue hardness/stiffness	^[^ ^132^ ^]^
	ZnO	Nanowire	Diameter: ≈150 nm Average length: ≈12 µm	Seed‐assisted hydrothermal synthesis	Detection of the urea/uric‐acid concentration	Human skin	Urea concentration range: 0–80 × 10^–3^ m, uric acid concentration range: 0–0.6 × 10^–3^ m	Linear response to urea concentration and uric acid concentration	^[^ ^174^ ^]^
	PVDF	Film	Thickness: ≈28 µm	\	Detection of tactile signals of submucosal tumors	Mucosa	Young's modulus range: 1.01–3.51 MPa	Sensor response is proportional to the Young's modulus of test sample	^[^ ^175^ ^]^
Bionic/Smart Devices	ZnO	Nanowire	Average diameter: ≈250 nm Average length: 10–14 µm	Seed‐assisted hydrothermal synthesis	Detection of taste‐producing substances	Taste bud	2 × 10^−2^ m ascorbic acid	171.7 relative response value	^[^ ^139^ ^]^
	ZnO	Nanowire	Diameter: ≈50 nm Aspect ratio: 20	Solution‐based hydrothermal synthesis	Detection of both static and dynamic tactile stimuli	Human skin	Within 0.3 kPa pressure	–6.8 kPa^−1^ sensitivity	^[^ ^88^ ^]^
	ZnO PVDF	Film	\	Thermal evaporation and wet‐chemical method	Detection of motion‐powered atmosphere	Human skin	57° bending angle, relative oxygen concentration range: 20%‐50%, relative humidity range: 45%‐85%	Linear response to oxygen concentration and relative humidity	^[^ ^137^ ^a]^
	PVDF	Film	\	\	Detection of plantar pressure signals	Human plantar	Within 200 kPa pressure	0.00814 V kPa^–1^ sensitivity	^[^ ^176^ ^]^
	PZT	Nanofiber	\	Electrospinning	Detection of tactile pressure	Human skin	Measurement pressure range: 0–1300 kPa	18.96 V kPa^–1^ sensitivity	^[^ ^177^ ^]^
	PVDF	Nanofiber	Average diameter: 993 ± 631 nm	Electrospinning	Sensing of pressure, integrating cold/heat	Human skin	Measurement force range: 3–53 N, measurement initial temperature range: 20–80 °C	Pressure‐sensing signal became steady‐state, while pyroelectric signal appeared as a pulse	^[^ ^138^ ^]^
	PZT P(VDF‐TrFE)	Film	Thickness: ≈100 µm	Hydrothermal method	Harvested and converted mechanical energy from human activities	\	<5 Hz bending frequency	≈16 V maximum output voltage	^[^ ^100^ ^]^
Cancer treatment	BaTiO_3_	Nanoparticle	Radius: ≈150 nm	\	Generated electrical stimulation to cancer cells	Cancer cell	1 MHz, 1 W cm^–2^ ultrasonic wave	Blocked the cell cycle of cancer cells and slowed their proliferation	^[^ ^27a^ ^]^
	Black phosphorus	Nanosheet	Average thickness: 5.3 ± 3.7 nm Average lateral dimension: 162.4 ± 99.4 nm	Ultrasonic exfoliation	Eliminated cancer cells with reactive oxygen species	Cancer cell	1 MHz, 1.5 W cm^–2^ ultrasonic wave	>70% cell viability losses of cancer cells	^[^ ^146^ ^]^
	P(VDF‐TrFE)	Nanoeel	\	Templated method	Magnetic manipulation for locomotion and pulsatile drug release	Cancer cell	5‐15 mT, 1–16 Hz magnetic field (locomotion) 10 mT, 7 Hz magnetic field (drug release)	≈35% cancer cell death	^[^ ^148^ ^]^
	P(VDF‐TrFE)	Nanowire	Average diameter: ≈250 nm	Templated method	Magnetic manipulation for locomotion and magnetoelectric drug release	Cancer cell	<10 mT rotating magnetic field (locomotion) Alternating magnetic field with same energy source (drug release)	≈40% cancer cell death	^[^ ^178^ ^]^
	BaTiO_3_	Nanoparticle	Average diameter: 106.91 ± 49.72 nm	Solvothermal process and thermal annealing	Eliminated cancer cells with reactive oxygen species	Cancer cell	1 W cm^–2^ ultrasonic wave	Cancer cell viability decreased to 12.6%	^[^ ^67b^ ^]^
	KNNSe	Ceramic	Diameter: ≈10 mm Thickness: ≈1 mm	Solid phase sintering	Eliminated cancer cells	Cancer cell	3 d of cocultivation	Cancer cell viability decreased to 30%	^[^ ^179^ ^]^
	PVDF	Film	\	\	Eliminated cancer cells	Cancer cell	12 d of intermittent continuous light stimulation	87.46% tumor inhibition rate	^[^ ^180^ ^]^
Tissue regeneration	ZnO	Nanorod	Length: 2.79 ± 0.14 µm Diameter: 0.58 ± 0.07 µm	Hydrothermal method	Generation of endogenous electric field at the wound	Skin wound	2 × 2 cm^2^ area with 95.2% ZnO nanorods filling density	Enhanced cell migration, metabolic activity, and differentiation	^[^ ^153^ ^]^
	BaTiO_3_	Nanoparticle	\	\	Regenerated bone	Osteoblast cell	60 N, 3 Hz force	Enhanced alkaline phosphatase activity and bone‐inducing activity	^[^ ^27b^ ^]^
	LiNbO_3_	\	\	\	Fabricated therapeutic vascular tissue	Skeletal muscle	4 weeks after transplantation	72.8% of blood flow restored	^[^ ^181^ ^]^
	PVDF	Nanocomposite	\	Solvent casting	Induced cellular mechano‐ and electro‐transduction process in bone	Osteoblast cell	4 d of cocultivation	Formed a bone‐mimicking structure that improves cell seeding and proliferation	^[^ ^151^ ^]^
	P(VDF‐TrFE)	Nanofiber	Diameter: 1.24 ± 0.13 µm	Electrospinning	Stimulated cardiac muscle cells	Cardiac muscle cell	12 d of cocultivation	Promoted cardiomyocyte attachment, proliferation and alignment, preserving contractility	^[^ ^182^ ^]^
	PVDF	Nanofiber	\	Electrospinning	Stimulated osteoblast cells	Osteoblast cell	3 d of cocultivation	Exhibited higher Saos‐2 cells activity	^[^ ^105^ ^]^
Neurotrauma and neurodegenerative treatment	P(VDF‐TrFE) BaTiO_3_	Film	\	\	Enhanced differentiation during cell growth	Neuronal cell	1 W cm^–2^ ultrasonic wave	Promoted neuronal maturation and neurite outgrowth in SH‐SY5Y	^[^ ^183^ ^]^
	BaTiO_3_	Nanoparticle	Diameter: 58 ± 15 nm	\	Induced differentiation of targeted neural stem‐like cell	Neural stem‐like cell	1 MHz, 1 W cm^–2^ ultrasonic wave	Achieved navigation to a specific neural stem‐like PC12 cell in a controllable way	^[^ ^159^ ^]^
	KNNS	Ceramic	\	Solid phase sintering	Stimulated retina	Retina	30 Vpp input voltage	≈72 µA current, ≈9.2 nA µm^–2^ current density	^[^ ^161^ ^]^
	PVDF	Nanocomposite	\	\	Stimulated differentiation and proliferation of neuronal cells	Neuronal cell	7 d of cocultivation	Showed higher PC12 cell activity	^[^ ^184^ ^]^
	PVDF	\	\	Solvent casting	Stimulated neuronal cells	Neuronal cell	4 months after being implanted	Exhibited significant electrophysiological, morphological and functional nerve restoration	^[^ ^185^ ^]^
	ZnO	Nanoparticle	Average diameter: 30–80 nm	\	Stimulated cell attachment and proliferation	Schwann cell	18 weeks in vivo	Improved severe nerve defect recovery	^[^ ^104^ ^]^
Antifouling treatment	BaTiO_3_	Nanoparticle	Average diameter: ≈130 nm	Hydrothermal method	Whitened teeth	Teeth	10 h of vibration	Exhibited a whitening effect	^[^ ^27c^ ^]^
	PVDF	Film	Thickness: 110 µm	\	Inhibited bacterial growth and antifouling effects	\	4 Hz stimuli	Inhibited biofilm formation effectively	^[^ ^166^ ^]^

**Figure 11:**
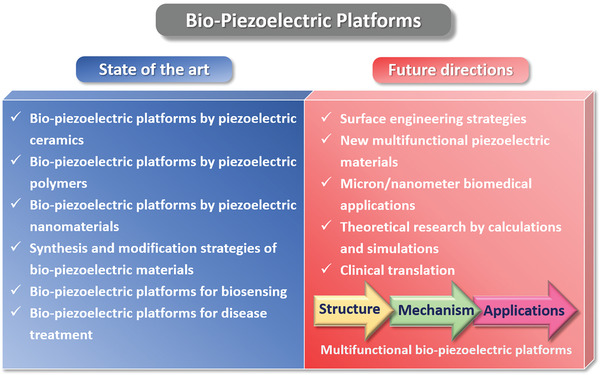
Summary of current status and future research directions of bio‐piezoelectric platforms.

Despite the significant application potential of piezoelectric materials in the field of biomedicine, a number of challenges in the development of bio‐piezoelectric platforms remain, as shown in **Figure** **11**.

(1) Bio‐piezoelectric platforms should fulfill critical biomedical requirements prior to their practical application. For applications in biosensing and implant devices, bio‐piezoelectric platforms are expected to possess high biocompatibility, excellent mechanical flexibility and high electromechanical conversion efficiency. For disease treatment with bio‐piezoelectric nanoplatforms, their biodegradability, immunogenicity and tissue accumulation must be also considered. Although significant effort has been paid to the development of bio‐piezoelectric materials, current available bio‐piezoelectric platforms are still limited in terms of the range of available materials and there is a need for research in the development of new piezoelectric and ferroelectric materials/composites and their surface modification. In this regard, the development of lead‐free ferroelectric ceramics, polymers, and composites could be particularly important.
(2) Most studies on bio‐piezoelectric materials have a focus on exploring and improving material physical properties, such as morphology, transparency, flexibility, piezoelectricity, and mechanical strength. Limited attention has focused on their chemical properties as well as their synergy with piezoelectricity. For example, many bio‐piezoelectric materials possess fascinating chemical properties in terms of photocatalysis and electrocatalysis, such as MoS_2_ and black phosphorus. We believe more innovative bio‐piezoelectric platforms will emerge when material piezoelectricity is combined in synergy with their chemical properties.
(3) At present, the development of theoretical calculations and simulations (such as density functional theory, molecular dynamics, molecular structure mechanics) has greatly promoted the progress of theoretical research. Such approaches can inform materials design, which can greatly accelerate the investigation of bio‐piezoelectric materials. Of particular interest is the potential for the use of high‐throughput (HT)^[^ ^168^ ^]^ and machine learning methods^[^ ^169^ ^]^ for materials discovery.
(4) Ultrasound is currently the main mechanical stimuli to induce material piezoelectricity. Nevertheless, a high dosage of ultrasound can lead to tissue damage as a result of ultrasonic cavitation effects. Therefore, it is desirable to develop additional biocompatible strategies to induce material piezoelectricity. In addition, improving the piezoelectricity of existing bio‐piezoelectric platforms can also minimize the level of applied ultrasonic power to a biologically safe range.
(5) Bio‐piezoelectric materials offer an excellent avenue to induce multifunctionality in the bioactive platforms. For instance, pyroelectric materials as a sub‐class of bio‐piezoelectrics have also shown potential for the application in biomedicine, especially for cancer photothermal therapy. These materials possess an inherent polarization which changes as the material is thermally excited, resulting in a change in the bound surface charge. This effect has been utilized to develop a pyroelectric dynamic therapy^[^ ^170^ ^]^ that generates reactive oxygen species from pyroelectric nanomaterials during thermal excitation by near‐infrared light. The reactive oxygen species attack the cancer cells and heat can shock proteins responsible for the thermotolerance of tumor cells resulting in an enhanced therapy efficacy. Since all pyroelectric materials and also piezoelectric, there is potential to use such materials during a combined ultrasound and photothermal therapy.
(6) Many bio‐piezoelectric platforms which are based on electrical stimulation to promote cell growth, differentiation and proliferation have been reported; however, the safe range of electrical signals has yet to be clearly defined. Studies have shown that an inappropriate electrical stimulation may have negative effect on cell growth and proliferation, and can even lead to cell death.^[^ ^171^ ^]^ Therefore, the safety of piezoelectric stimulation to different cells and tissues should be thoroughly evaluated and carefully explored. In addition, piezoelectric stimulation is influenced by a variety of parameters, such as piezoelectric coefficient, material morphology, surface charge, duration of applied mechanical stress, and site of action. Based on these aspects, the electrical stimulation generated by piezoelectric materials is expected to be precisely controlled to achieve desired therapeutic effects.
(7) Clinical application is the ultimate goal of the bio‐piezoelectric platforms. Although bio‐piezoelectric materials have achieved preliminary success in biomedical field, their long‐term toxicity, targeting ability, biosafety, and biodegradability should be further assessed. To minimize the required dosage, the development of bio‐piezoelectric materials with high piezoelectric efficiency is highly desirable. Moreover, optimizing synthesis methods and developing new surface engineering technologies are of significance for promoting the clinical translation of bio‐piezoelectric platforms.

While bio‐piezoelectric platforms are in their infancy stage, there continues to be plenty of scope and opportunities for research and development in this intriguing growing area. With a broad interdisciplinary research effort in the pursuit of new bio‐piezoelectric materials and systems, it can be envisioned that these new emerging platforms will achieve exciting biomedical applications in the future.

## Conflict of Interest

The authors declare no conflict of interest.
